# Scouter predicts transcriptional responses to genetic perturbations with large language model embeddings

**DOI:** 10.1038/s43588-025-00912-8

**Published:** 2025-12-05

**Authors:** Ouyang Zhu, Jun Li

**Affiliations:** Department of Applied and Computational Mathematics and Statistics, University of Notre Dame, Notre Dame, IN USA

**Keywords:** Statistical methods, High-throughput screening, Genetics

## Abstract

Gene perturbation experiments followed by transcriptomic profiling are vital for uncovering causal gene effects. However, their limited throughput leaves many perturbations of interest unexplored. Computational methods are therefore needed to predict genome-wide transcriptional responses to gene perturbations that were not experimentally assayed within a given dataset. Existing approaches often rely on Gene Ontology graphs to encode prior knowledge, but their predictive power and applicability are constrained by the graphs’ sparsity and incomplete gene coverage. Here we present Scouter, a computational method that uses gene embeddings generated by large language models and a lightweight compressor–generator neural network. Scouter accurately predicts transcriptional responses to both single- and two-gene perturbations, reducing errors from state-of-the-art Gene Ontology-term-based methods (GEARS and biolord) by half or more. Unlike recent approaches based on fine-tuning gene expression foundation models, Scouter offers substantially better accuracy and greater accessibility; it requires no pretraining and runs efficiently on standard hardware.

## Abstract (web summary)

A lightweight AI method called Scouter that predicts genome-wide transcriptional responses to single- and two-gene perturbations using large language model embeddings is presented and achieves substantially higher accuracy than leading approaches.

## Main

**Fig. 1:**
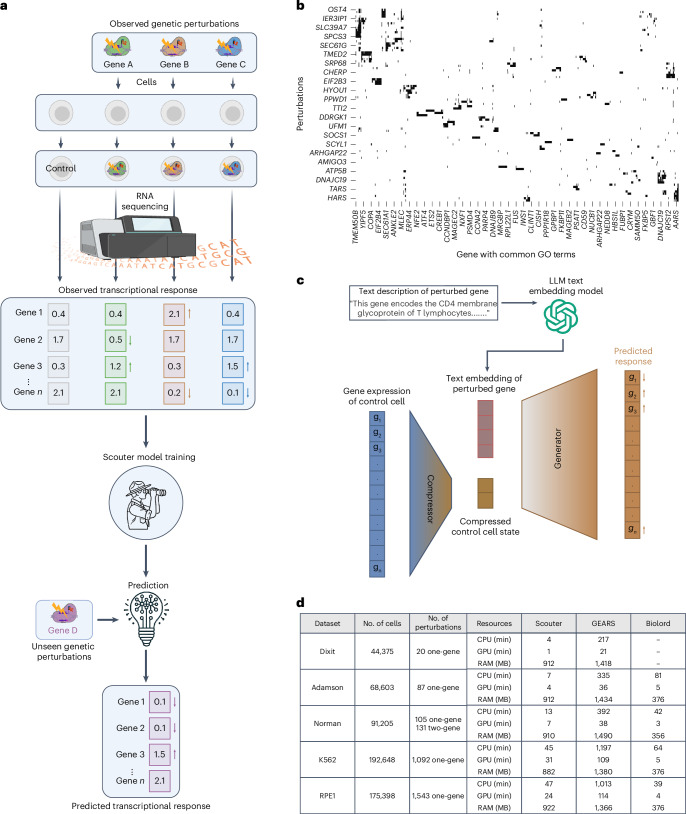
The problem that Scouter addresses, the architecture of Scouter and the datasets used. **a**, Perturbation experiments provide gene expression data for multiple single-gene perturbations. Methods such as Scouter learn from observed transcriptional responses and predict the responses of unseen perturbations. Single-gene perturbations are used for this illustration, but Scouter is applicable to both single- and two-gene perturbations. The upward and downward arrows indicate up- and down-regulation of gene expression, respectively. *n* denotes the total number of genes. **b**, A binary heatmap of the gene embedding matrix derived from GO terms. Each row corresponds to a gene perturbed in the Adamson dataset that has at least one GO term, while each column corresponds to a gene that shares at least one GO term with another gene in the row. The matrix includes 82 perturbations × 635 genes, with axis ticks automatically sampled for visual clarity. Cells are colored white to indicate zero values and black for non-zero values. The predominance of zero entries indicates minimal overlap in GO annotations among the genes. **c**, The Scouter architecture based on a compressor–generator neural network. *g*_1_, *g*_2_, …, *g*_*n*_ represent the observed or predicted expression levels of the *n* genes. **d**, A summary of the datasets used and the computational time and resources required by different methods. Each dataset contains about 5,000 genes. Statistics for biolord on the Dixit data are not reported as its predictions did not yield meaningful improvement over the baseline in our evaluations. Source data

Gene perturbation experiments play a crucial role in biological and biomedical research owing to their potential to reveal causal relationships and their direct medical and translational implications^1,2^. As illustrated in Fig. 1a, perturbing a gene—via knockout, enhancement or suppression—typically alters the expression of many other genes owing to intricate gene interactions and regulatory dynamics. These changes can be quantified using techniques such as Perturb-seq^3^. However, high costs and practical constraints typically restrict the number of genes that can be individually perturbed in these experiments. Consequently, there is strong interest in using computational methods to predict the transcriptional outcomes of perturbations on other genes as if they had been experimentally manipulated in the same study.

The aim of this study is to develop a computational method to extrapolate from an experiment involving a limited number of gene perturbations (‘seen’ perturbations) to predict responses for perturbing other genes (‘unseen’ perturbations) within the same dataset or cell type. The challenge of predicting the outcomes of unseen perturbations (as illustrated in Fig. 1a) is substantial. The primary issue is the high dimensionality of the desired prediction outcome—the expression levels of all genes—in contrast to the simplicity of the input: the identity of the perturbed gene, which is a single categorical variable. Although one could incorporate the expression data from an unperturbed (control) cell as input, this information remains constant across all perturbations in the experiment. The critical variable, the identity of the perturbed gene, remains singular and poses a major challenge for encoding. Traditional methods such as one-hot encoding are entirely unsuitable as they fail to capture the intrinsic characteristics of genes or the relational dynamics among them.

To address these challenges, any feasible algorithm must account for prior knowledge of gene–gene interactions and represent an unseen gene within the same space as seen genes. The currently available algorithms, GEARS^4^ and biolord^5^, both utilize gene regulatory networks informed by the Gene Ontology (GO) graph^6^, where nodes represent genes and edges are weighted by the number of shared GO terms. These algorithms represent each perturbed gene by a combination of its nearest neighbors in the graph.

Despite these advancements, the reliance on the GO graph presents substantial limitations. Firstly, the GO graph is highly sparse (as illustrated in Fig. 1b), meaning that only a very small fraction of gene pairs share GO terms, which can compromise the accuracy and reliability of predictions. Secondly, the exclusion of some genes from the GO graph precludes predicting outcomes for perturbations involving these genes. Consequently, GEARS and biolord are unable to predict outcomes for all genes (see Supplementary Section 1 for a detailed discussion). Lastly, the use and extensibility of these algorithms are constrained by the specialized nature of machine learning techniques required for graph data.

## Results

### Overview of the Scouter framework

In this paper, we propose a computational method called Scouter, short for a transcriptional response predictor for unseen genetic perturbtions with large-language model (LLM) embeddings, that overcomes previous limitations and markedly enhances the prediction accuracy. Scouter employs a fundamentally different approach to capture gene–gene interactions: the embeddings of genes generated by pretrained LLMs. These embeddings, represented as long and dense numeric vectors, have been demonstrated in recent studies to capture complex regulatory relationships between genes more efficiently than traditional graphical models^7–12^. Specifically, the gene embeddings used by Scouter, each of length 1,536, were sourced from the GenePT^11^ paper. The generation of these embeddings did not require users to train their own models. Instead, they were produced at very low cost by utilizing OpenAI/ChatGPT’s ‘text-embedding-ada-002’ model, which converts textual descriptions of genes into embeddings. These descriptions, such as the official National Center for Biotechnology Information (NCBI) gene descriptions, are rich in information about gene regulations, functions and structures (see Supplementary Section 2 for a detailed discussion).

Scouter incorporates both the expression of all genes in the control cell and the ChatGPT embedding of the perturbed gene as input to a neural network that follows a compressor–generator framework, depicted in Fig. 1c. Inspired by the concept of ‘disentanglement’ introduced by Gabbay and Hoshen^13^, Scouter first compresses the gene expression of the control cell into a short and dense vector. This vector is then concatenated with the embedding of the perturbed gene—which remains fixed and is not updated during training—and the resulting combined vector is passed to the generator to produce the predicted perturbation response.

Scouter is evaluated on the five Perturb-seq datasets selected and preprocessed in the GEARS paper: Dixit^3^, Adamson^14^, Norman^15^, Replogle K562^16^ and Replogle RPE1^16^. The basic information of these datasets is provided in Fig. 1d. These datasets each contain more than 40,000 cells, but the number of different perturbations can be as few as 20. This scenario makes training the model tricky: if one averages the expression profiles of all control cells to use as the input and similarly averages the profiles of all cells with the same gene perturbation as the output, the training may suffer severely owing to an insufficient sample size. Therefore, unlike GEARS and biolord, Scouter randomly selects a control cell and a perturbed cell, using them as the network’s input and output for training, respectively. This approach, given *n*_0_ control cells in the data and *n*_*k*_ cells with gene *k* perturbed, yields a total number of training samples equal to *n*_0_∑_*k*_*n*_*k*_, which is numerous. This strategy, combined with Scouter’s relatively simple network architecture, allows it to train effectively even on the challenging Dixit dataset.

Furthermore, despite the increased training sample size, Scouter maintains a reasonable memory footprint and demonstrates computational times comparable to those of biolord, both of which are more efficient than GEARS. Detailed metrics are presented in Fig. 1d.

### Benchmarking the predictive performance for single- and two-gene perturbations

**Fig. 2:**
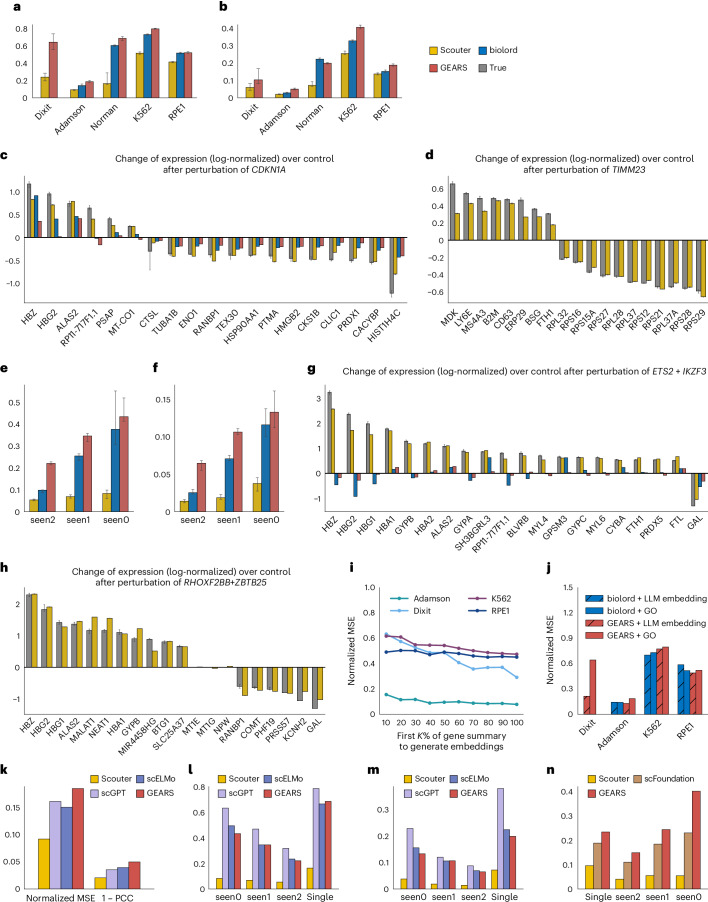
Performance of different methods on real datasets. **a**,**b**, The normalized MSE (**a**) and 1 − PCC (**b**, bar heights) with 50% bootstrap confidence intervals (whiskers) of Scouter, biolord and GEARS for single-gene perturbations. Biolord’s results for the Dixit dataset are omitted as its predictions did not provide meaningful improvement over the baseline. **c**, An example of true and predicted expression changes relative to control for a single-gene perturbation. The median of the predicted changes (bar heights) with 50% bootstrap confidence intervals (whiskers) from Scouter, biolord and GEARS are shown. **d**, An example of true and predicted expression changes relative to control for a single-gene perturbation not present in the GO graph; biolord and GEARS are unable to generate predictions. **e**,**f**, The mormalized MSE (**e**) and 1 − PCC (**f**) of Scouter, biolord and GEARS for two-gene perturbations. **g**, An example of true and predicted expression changes relative to control for a two-gene perturbation. Predictions from Scouter, biolord and GEARS are shown. **h**, An example of true and predicted expression changes relative to control for a two-gene perturbation. Biolord and GEARS are unable to generate predictions owing to limited GO graph coverage. **i**, The effect of truncating NCBI summaries to the first *K*% of words on Scouter’s performance. This plot shows how the normalized MSE varies as progressively shorter portions of each gene’s textual summary are used to generate embeddings. **j**, The performance of GEARS and biolord when replacing GO-based embeddings with LLM-derived embeddings. The bar chart reports the normalized MSE across four single-gene perturbation datasets. ‘+LLM embedding’ bars use LLM-derived embeddings; ‘+GO’ bars use GO-based embeddings. **k**–**m**, Benchmarking Scouter against gene-expression foundation models: the Normalized MSE and 1 − PCC on the Adamson dataset (**k**), and the Normalized MSE (**l**) and 1 − PCC (**m**) on the Norman dataset, stratified by perturbation subgroup: seen0, seen1, seen2 and single. **n**, A comparison between Scouter, scFoundation and GEARS on the Norman dataset using the raw (not normalized) MSE, broken down by perturbation subgroup. Source data

To quantitatively evaluate the results of different methods, we adopt the metrics used by GEARS and biolord (see Methods for detailed definitions): the normalized mean squared error (MSE) and one minus the normalized Pearson correlation coefficient (1 − PCC) across the top 20 differentially expressed genes (DEGs). Both metrics favor small values. Figure 2a,b show the median (height of the bar) and the 50% confidence interval (ticks on the top of the bar) of these two metrics for each method across the five datasets. Clearly, Scouter achieves the lowest MSE and 1 − PCC values for all five datasets, with substantial improvement over the other two methods: on average, Scouter’s MSE and 1 − PCC values are only about half of those of biolord (48.9% and 56.0%) and GEARS (51.0% and 54.1%).

Figure 2c showcases the true versus predicted expression profiles of the top 20 DEGs following perturbation of the gene *CDKN1A*. Scouter’s predictions are more accurate than those of biolord and GEARS for 18 out of 20 genes (90%). Figure 2d focuses on the perturbation of the gene *TIMM23*, which is absent from the GO graph, thus rendering biolord and GEARS unable to provide predictions. In contrast, Scouter continues to deliver precise predictions for the majority of these top DEGs.

Moreover, Scouter is capable of predicting transcriptional responses from the simultaneous perturbation of two genes. This functionality is implemented by summing the ChatGPT embeddings of the two genes and using the resultant vector as the input embedding. Figure 2e,f shows the MSE and 1 − PCC for the Norman dataset, which is the sole dataset among the five that includes two-gene perturbations. Following biolord and GEARS, the predictions are categorized on the basis of the number among the two genes that were perturbed in the training data: seen2 (both perturbed, but not simultaneously), seen1 (only one perturbed) and seen0 (neither perturbed). Scouter substantially outperforms biolord and GEARS, with MSE and 1 − PCC values that are less than a half (34.7% and 38.2%) of those of biolord and less than a quarter (21.2% and 22.6%) of those of GEARS. Scouter’s performance is even more pronounced in two-gene perturbation cases compared to single-gene cases.

Figure 2g presents results for the dual perturbation of *ETS2* and *IKZF3*, where Scouter accurately predicts the expression of all 20 genes. In contrast, both biolord and GEARS not only fail to predict the magnitude of the changes accurately but also make numerous errors in the direction of the changes. Figure 2h provides an example of a dual perturbation where the other models cannot make predictions owing to limited GO graph coverage. Here, Scouter still offers accurate predictions for almost all genes.

Owing to complex genetic interactions, the response to a two-gene perturbation can often be markedly different from the sum of the responses of their individual perturbations. These interactions may be classified into subtypes such as synergy, suppression, redundancy, neomorphism and epistasis^4,15^. GEARS has demonstrated impressive capabilities in predicting these various subtypes. We have also shown that Scouter outperforms GEARS in this regard. For more details, see Supplementary Section 3.

## Discussion

The efficacy of Scouter probably stems from the interplay of several key factors. First is the utilization of high-dimensional gene embeddings from LLMs, which are dense vectors packed with comprehensive information, including but not limited to gene–gene interactions (Fig. 2i; see Supplementary Section 4 for details). Second, its compressor–generator network architecture effectively leverages the rich data in the gene embeddings. In tests, the use of LLM gene embeddings as input for GEARS and biolord resulted in only modest improvements, indicating their lower efficiency in utilizing such information (Fig. 2j; see Supplementary Section 5 for details). Finally, Scouter’s training strategy, which involves random selections of control and perturbed cell pairs, is pivotal for successful training.

Our primary benchmarks focus on GEARS and biolord, which—like Scouter—are purpose-built models specifically designed to predict transcriptional responses to unseen gene perturbations. However, given the growing interest in adapting gene expression foundation models for this task, we also conducted additional experiments to benchmark Scouter against several such models—scGPT^9^, scELMo^17^ and scFoundation^10^—which were pretrained by their authors on large collections of scRNA-seq datasets and fine-tuned by us for perturbation prediction. Across both the Adamson and Norman datasets, Scouter consistently outperforms these foundation models by large margins in both single-gene and two-gene perturbation scenarios. Summary statistics are shown in Fig. 2k–n. In addition to its substantial gains in predictive accuracy, Scouter offers superior accessibility: it can be trained end-to-end on a single Perturb-seq dataset using minimal computational resources—a modest graphics processing unit (GPU) (for instance, an A40 or lower) or even a central processing unit (CPU) (for instance, the Apple M2 chip in a MacBook Pro)—and completes training in under 1 h even on the largest dataset we evaluated. See Supplementary Section 6 for detailed results and a systematic discussion of Scouter’s advantages over foundation model-based approaches.

For further discussion on extensions of Scouter using cell type-aware gene embeddings and cross-condition prediction, refer to Supplementary Sections 7 and 8.

## Methods

### Overview of Scouter

Scouter is a deep neural network that extrapolates from a gene perturbation experiment such as Perturb-seq, which involves a small number of perturbations, to predict responses to unseen perturbations in the same biological condition or cell type. These experiments typically also include ‘control cells’, which are cells that are not perturbed.

To use Scouter, the user provides a gene perturbation dataset in which each cell is labeled with the perturbation it received (that is, the targeted gene or ‘control’ if unperturbed). Scouter is then trained on this dataset, holding out a subset of perturbations for validation and parameter tuning. Once trained, the model can be used to predict transcriptional outcomes for perturbations not observed in the training set. These predictions can help guide experimental design by prioritizing candidate perturbations for future validation or follow-up studies.

Scouter inputs the expression profile of a control cell and the LLM-based embedding of the perturbed gene, then outputs the predicted expression profile of the perturbed cell. The following subsections cover how the gene embeddings are generated (‘LLM embeddings of gene text description’ section), the design of Scouter’s network architecture (‘Network architecture of Scouter’ and ‘Loss function of Scouter’ sections) and the training of Scouter’s network (‘Data splits’ and ‘Selection of hyperparameters’ sections).

### LLM embeddings of gene text description

Predicting responses to unseen genetic perturbations represents a classical task of extrapolation in categorical variables, which is inherently challenging owing to the lack of inherent order or distance among these variables. This makes it difficult for models to generalize from known to unknown genes. Motivated by the demonstrated effectiveness of LLMs in genomic research^11,12^, we propose utilizing embeddings of gene textual descriptions. By transforming the rich semantic content of textual descriptions into continuous vector representations with meaningful distances, we aim to capture semantic relationships between genes. This method allows the model to infer properties of unseen genes on the basis of their descriptions, offering a scalable solution to the challenge of extrapolation.

The LLM embeddings considered in this work were provided in GenePT^11^, which employs NCBI text descriptions of each gene, and OpenAI’s ‘text-embedding-ada-002’ model to generate the corresponding LLM embeddings^18^ with a length of 1,536.

The LLM embeddings of perturbed genes, which serve as part of the inputs to Scouter, remain unchanged during training.

### Network architecture of Scouter

Scouter is a deep neural network model with a simple architecture that facilitates easy training. For a dataset containing *n* cells, with *n*_0_ control cells and *n* − *n*_0_ perturbation cells, each training epoch for Scouter processes *n* − *n*_0_ triplets: $D={\{\mathbf{x}_{i},\mathbf{E}({p}_{i}),\mathbf{{c}}_{i}\}}_{i = 1}^{n-{n}_{0}}$. Here **x**_*i*_ is the gene expression vector of perturbed cell *i*, *p*_*i*_ represents the perturbed gene in cell *i*, **E**(*p*_*i*_) denotes the embedding vector of *p*_*i*_ and **c**_*i*_ is the gene expression vector of a randomly selected control cell.

Scouter incorporates a compressor *C* and a generator *D* (Fig. 1). Initially, *C* condenses the highly sparse vector **c**_*i*_ into a compact cell state **S**_*i*_ using a narrow bottleneck. Subsequently, **S**_*i*_ and **E**(*p*_*i*_) are concatenated and provided to *D*, which then generates the corresponding transcriptional response $\mathbf{\hat{x}}_{i}=D(\mathbf{S}_{i}\oplus {\mathbf{E}}({p}_{i}))$, where ⊕ denotes concatenation.

To predict the response to a perturbation on gene *g*, Scouter takes as input the embedding **E**(*g*) and *K* random control cells ${\{\mathbf{c}_{i}\}}_{i = 1}^{K}$ (with *K* set to 300) and returns ${\{\mathbf{\hat{x}}_{i}\}}_{i = 1}^{K}$. If a single point estimate is desired, users may compute the mean vector $\frac{1}{K}\mathop{\sum }\nolimits_{i = 1}^{K}\mathbf{\hat{x}}_{i}$ as a summary. To evaluate the robustness of this sampling-based prediction strategy, we performed a sensitivity analysis on the Adamson dataset (see Supplementary Section 9), varying both *K* and the random seed. The results confirm that Scouter’s performance remains highly stable across different values of *K* and random seeds.

### Loss function of Scouter

Scouter is trained using a loss function known as the autofocus direction-aware loss, initially proposed by GEARS. This loss function comprises two components: the autofocus loss and the direction-aware loss. Consider a batch of *N* triplets $D={\{\mathbf{x}_{i},\mathbf{E}({p}_{i}),\mathbf{c}_{i}\}}_{i = 1}^{N}$, which includes *M* unique perturbations *t*_1_, *t*_2_, …, *t*_*M*_. The autofocus loss is defined as

$${L}_{{\rm{autofocus}}}=\frac{1}{M}\mathop{\sum }\limits_{m=1}^{M}\frac{1}{| {N}_{{t}_{m}}| \times | G| }\sum _{i\in {N}_{{t}_{m}}}{(\mathbf{x}_{i}-\mathbf{\hat{x}}_{i})}^{2+\gamma },$$

where $| {N}_{{t}_{m}}|$ denotes the number of triplets with perturbation *t*_*m*_ and ∣*G*∣ is the length of the vectors **x**_*i*_ or $\mathbf{\hat{x}}_{i}$. This loss penalizes discrepancies between the predicted response vector $\mathbf{\hat{x}}_{i}$ and the observed response vector **x**_*i*_. The parameter *γ* adjusts the focus of the penalty on larger discrepancies. Meanwhile, the direction-aware loss is defined as

$${L}_{{\rm{direction}}}=\frac{1}{M}\mathop{\sum }\limits_{m=1}^{M}\frac{1}{| {N}_{{t}_{m}}| \times | G| }\sum _{i\in {N}_{{t}_{m}}}{[{\rm{sign}}(\mathbf{x}_{i}-\mathbf{c}_{i})-{\rm{sign}}(\mathbf{\hat{x}}_{i}-\mathbf{c}_{i})]}^{2}.$$

This component imposes additional penalties for errors in predicting the direction of changes. The total loss is then expressed as *L* = *L*_autofocus_ + *λL*_direction_, where *λ* is the weighting factor for direction awareness.

### Detailed architecture

Scouter consists of two neural modules: a compressor network that encodes the control cell state and a generator network that produces the predicted transcriptional response given the encoded control cell state and the LLM-based embedding of the perturbed gene. The compressor is implemented as a multilayer perceptron (MLP) with two hidden layers of size 2,048 and 512, followed by a bottleneck layer of size 64. The generator is a separate MLP with one hidden layer of size 2,048 and an output layer matching the input gene expression dimensionality. Both modules use scaled exponential linear unit activation functions after each hidden layer. Dropout is disabled in all layers (that is, the dropout rate is set to 0), although the network architecture supports optional AlphaDropout. Batch normalization is enabled after each hidden layer, while layer normalization is disabled. The gene embedding layer is fixed (nontrainable), with dimensionality 1,536, and is shared across all perturbation conditions.

### Training configuration

All models are trained using the Adam optimizer with an initial learning rate selected from {0.001, 0.005, 0.01} via grid search. An exponential learning rate decay is applied at each epoch with a decay factor *γ* = 0.9. The batch size is fixed at 256 across all datasets. Models are trained for a maximum of 40 epochs, with early stopping applied on the basis of validation loss improvement, using a patience of five epochs and a minimal improvement threshold of 0.001. The loss function is the autofocus direction-aware loss proposed in GEARS, consisting of a power-scaled MSE term and a direction consistency penalty. The exponent *γ* and weighting factor *λ* are selected via grid search from {0, 2} and {0.01, 0.05, 0.1, 0.5}, respectively. Gradient clipping with a max-norm of 1.0 is applied to stabilize training. During training, the model state with the lowest validation loss is retained and restored at the end of training.

### Selection of hyperparameters

To identify optimal hyperparameters for each dataset, we conducted a grid search based on the average performance on validation sets across all data splits. The architecture and training configurations—including network depth, layer sizes, batch size, dropout rate and learning rate decay—were fixed across all datasets as described above. Grid search was applied to a small number of remaining hyperparameters: the exponent *γ* in the autofocus loss, the direction-aware weighting factor *λ* and the initial learning rate.

The final hyperparameters selected for each dataset are as follows: For all datasets, we set *γ* = 0. For the Dixit dataset, we used *λ* = 0.05 and a learning rate of 0.01. For Adamson, we used *λ* = 0.01 and a learning rate of 0.001. For Norman, we used *λ* = 0.05 and a learning rate of 0.001. For Replogle K562 and Replogle RPE1, we used *λ* = 0.5 and a learning rate of 0.001.

### Data splits

Scouter employs a consistent network architecture yet is trained on each dataset individually to account for their inherent differences. Consequently, for the five datasets discussed herein, we trained five distinct Scouter models. Following the approach used in GEARS and biolord, we generated five different train–validation–test splits for the Adamson, Norman, Replogle K562 and Replogle RPE1 datasets, with 20% of the data set aside for testing and the remaining 80% split into 90% for training and 10% for validation. Owing to the small number of perturbations in the Dixit dataset, we generated ten different splits using an 80:10:10 train–validation–test ratio. For each dataset, we used the validation set for early stopping and hyperparameter tuning and evaluated the model’s performance as the average across all test sets. Note that these data splits ensure that the training and validation data do not include the perturbations whose responses are to be predicted (that is, the test data).

### Benchmarks

We compared Scouter’s performance to that of biolord and GEARS. The evaluation was conducted using the settings provided in their reproducibility repositories (refs. ^19^ and ^20^, respectively). Note that, although we meticulously followed the script code provided by biolord and GEARS, the exact values for the performance metrics, as illustrated in Fig. 2, may still differ from those reported in their papers. This discrepancy is probably due to variations or updates in the software versions.

To benchmark Scouter against gene expression foundation models—scGPT, scELMo and scFoundation—we followed the official pipelines provided by their authors (refs. ^21^, ^22^ and ^23^, respectively) and evaluated all models on the Adamson and Norman datasets.

Lastly, in Supplementary Section 10, we also compare Scouter with two heuristic baselines that do not utilize LLM embeddings. The results consistently demonstrate Scouter’s superior performance.

### Metrics of prediction performance

Owing to the fact that many genes do not exhibit significant differences between control and perturbed states, GEARS and biolord quantified model performance using the normalized MSE and normalized PCC across the top 20 DEGs. Given the expression vectors of a perturbed cell and a control cell, denoted as **x**_*i*_ and **c**_*i*_, respectively, and the corresponding prediction $\mathbf{\hat{x}}_{i}$, one has

$$\begin{array}{rcl}{\rm{normalized}}\,{\rm{MSE}}&=&{\rm{MSE}}(\mathbf{x}_{i},\mathbf{\hat{x}}_{i})/{\rm{MSE}}(\mathbf{x}_{i},\mathbf{c}_{i}),\\ {\rm{normalized}}\,{\rm{PCC}}&=&{\rm{PCC}}(\mathbf{x}_{i}-\mathbf{c}_{i},\mathbf{\hat{x}}_{i}-\mathbf{c}_{i}).\end{array}$$

We adopted the same criteria, with the exception that we used 1 − normalized PCC instead of the normalized PCC, so that both criteria favor smaller values.

Our evaluation follows the standard benchmarking convention used by GEARS and biolord, in which metrics are computed over the top 20 DEGs. In Supplementary Section 11, we extend this analysis to larger DEG sets and find that Scouter’s performance advantage remains consistently strong.

Moreover, to evaluate how well Scouter’s predicted expression profiles capture global transcriptional shifts, we computed a normalized energy distance ratio that compares the predicted and true perturbation distributions relative to control cells. Detailed results are provided in Supplementary Section 12.

### Computing resources

We measured the maximum random access memory (RAM) usage of each method using the nvidia-smi command. For CPU runtime measurements, a MacBook Pro (2022) equipped with 16 GB of RAM and an Apple M2 chip (eight-core CPU) was utilized. GPU runtime measurements were conducted on a server outfitted with a 32-core Intel Xeon Gold 6326 CPU, 256 GB of RAM and an NVIDIA A40 GPU, which possesses 48 GB of GPU memory.

### Reporting summary

Further information on research design is available in the Nature Portfolio Reporting Summary linked to this article.

## Supplementary information

Supplementary Information Supplementary Discussion, Figs. 1–17 and Tables 1–3.

Reporting Summary

## Source data

Source Data Fig. 1. Numerical source data for Fig. 1.

Source Data Fig. 2. Numerical source data for Fig. 2.

## Data Availability

No new data were generated for this study. All analyses used previously published, publicly available datasets. All datasets were obtained through the GEARS package, which provides preprocessed and thoroughly annotated data. The original datasets can be accessed via the Gene Expression Omnibus under accession nos. GSE90063 (Dixit), GSE90546 (Adamson), GSE133344 (Norman) and GSE146194 (Replogle K562 and Replogle RPE1). The GenePT gene embeddings are available via GitHub at https://github.com/yiqunchen/GenePT. Source data are provided with this manuscript.

## Code availability

Scouter is available as a Python package via GitHub at https://github.com/PancakeZoy/scouter. Code for reproducibility is available via GitHub at https://github.com/PancakeZoy/scouter_misc and via Zenodo at https://github.com/yiqunchen/GenePT (ref. ^24^), which includes well-documented scripts for obtaining data, training models, making predictions, evaluating performance and conducting comparisons.
