# Supplementary material for: Scouter predicts transcriptional responses to genetic perturbations with large language model embeddings
Source: Nat Comput Sci. 2025 Dec 5;6(1):21–8. doi: 10.1038/s43588-025-00912-8 (PMC12855003; doi:10.1038/s43588-025-00912-8)
Supplement: Supplementary file 1 — Supplementary Discussion, Figs. 1–17 and Tables 1–3. [file 43588_2025_912_MOESM1_ESM.pdf]

nature computational science

Supplementary information

https://doi.org/10.1038/s43588-025-00912-8

Scouter predicts transcriptional responses to genetic perturbations with large language model embeddings

In the format provided by the authors and unedited

Contents

1 Limitations of GO-term-based embeddings 2
2 Rich information in LLM-based embeddings 3
3 Scouter accurately predicts types of genetic interactions 5
4 Ablation study: the performance of Scouter with degraded LLM-based embeddings 10
5 Using LLM-based embeddings for GEARS and biolord 13
6 Comparison with gene-expression foundation models 14
7 Exploring cell-type-specific gene embeddings 18
8 Scouter's performance on cross-dataset prediction 20
9 Robustness of prediction to control cell sampling 22
10 Additional baselines 22
11 Robustness of predictive accuracy across varying numbers of DEGs 24
12 Assessing distributional accuracy with energy distance 24
Supplementary Tables 29
Supplementary Figures 32

1 Limitations of GO-term-based embeddings

Machine learning predictions typically require numeric values as inputs. The question then arises: how do we numerically represent a perturbed gene using a numeric vector? This vector must capture the characteristics of the gene, and when compared with vectors from other genes, it should also delineate their similarities, differences, and various relationships.

A naive approach to representing a gene is through one-hot encoding. Both GEARS and biolord, on the other hand, utilize embeddings derived from Gene Ontology (GO)–based networks [1]. In the following sections, we will explore the serious limitations of these two approaches. We will then explain how our LLM-based gene embeddings overcome these limitations, offering a more comprehensive representation of genes and their relationships.

One-hot encoding is entirely unsuitable for our task. It assigns to each gene a vector that contains a single "1" and zeroes everywhere else. For instance, in a scenario with four genes, one-hot encoding would assign the vector (1, 0, 0, 0) to gene 1, (0, 1, 0, 0) to gene 2, (0, 0, 1, 0) to gene 3, and (0, 0, 0, 1) to gene 4. This method fails to capture any intrinsic characteristics of the genes. Moreover, the similarity between any pair of genes, which could be measured by the inner product of their vectors, is always zero. This demonstrates that one-hot encoding does not capture any similarities or other relational dynamics between genes.

The gene embeddings derived from GO-based networks, as used in GEARS [2] and biolord [3], capture gene-gene similarities reflected in their shared GO terms. Here, we briefly describe how these embeddings are defined. Initially, the GO graph is constructed as a bipartite network that connects genes to pathway GO terms. In this graph, an edge exists between a gene and a GO term if the gene is annotated with that specific term. Let N_{GO} denote the entire set of genes in this graph, with $|N_{GO}|$ representing the total number of genes in the set N_{GO}. For each gene i, N_i denotes the set of GO terms associated with it. To quantify the similarity between any two genes, i and j, the the Jaccard index is employed. It is defined as: $J_{i,j} = \frac{|N_i \cap N_j|}{|N_i \cup N_j|}$. This index measures the proportion of shared GO terms between the two genes relative to the total unique GO terms they encompass. Subsequently, an embedding for each gene x is constructed by calculating its Jaccard similarity with every other gene in the graph, resulting in a vector of length $|N_{GO}|$:

$$\boldsymbol{v}_x = (J_{x,1}, J_{x,2}, \cdots, J_{x,|N_{GO}|}) \tag{1}$$

Theoretically, this vector representation can capture known relationships between genes as defined by the GO terms. However, it has significant limitations.

First of all, GO graph does not cover all genes. In the studies of GEARS and biolord, $|N_{GO}| = 9976$, which is significantly fewer than the total number of genes in the genome. As a result, many genes are not represented in N_{GO}, and their perturbations cannot be predicted. In fact, even among the limited number of genes experimentally perturbed in the Dixit, Adamson, Norman, K562, and RPE1 experiments, 1, 5, 7, 5, and 9 genes, respectively, are not in the GO graph.

Another, more significant limitation of GO-term-based embeddings is their inherent sparsity. Even for genes included in the GO graph, most do not share any GO annotations. As a result, the embeddings, $\boldsymbol{v}_x$, are predominantly composed of zeros. For example, in the Adamson dataset, where 82 perturbed genes have GO annotations, the initial embedding matrix has dimensions 82×9976. Yet, 9341 out of the 9976 columns contain only zeros. Removing these all-zero columns reduces the matrix to just 635 columns with at least one nonzero value. However, even this reduced matrix $E \in \mathbb{R}^{82 \times 635}$ remains highly sparse. To illustrate, we plot a heatmap of E in Figure 1b of the main text, where white represents zeros and black represents non-zeros. This heatmap is clearly highly sparse: the average overlap (the mean number of shared nonzero entries between any two gene vectors) is merely 0.58 entry out of the 635 possible. This extreme sparsity significantly limits the embeddings' effectiveness in capturing the full spectrum of gene-gene relationships.

2 Rich information in LLM-based embeddings

LLM embeddings overcome the limitations of GO-term-based embeddings and offer compelling advantages. First, LLM embeddings encompass a much broader range of genes: as long as a gene is accompanied by a well-written and biologically accurate textual description, an embedding can always be obtained using an LLM. The GenePT embedding matrix we utilized covers 33,985 genes, significantly exceeding the number of genes represented by $|N_{GO}|$.

Second, unlike the sparse GO-term-based embeddings, LLM embeddings are dense, meaning all entries in the vector are non-zero, real-valued numbers. These embeddings encapsulate a wealth of gene information not confined to GO terms. LLM embeddings are generated from NCBI descriptions of genes [4], which are manually curated and encompass detailed information about gene functions, structures, roles in biological pathways, and regulatory mechanisms—substantially richer than what is provided by GO terms alone. Powerful LLMs like those from OpenAI can effectively compress this extensive information into a dense vector embedding. This embedding serves as an input for Scouter. The efficacy

of LLMs in condensing complex information into vector embeddings has been demonstrated across numerous applications in recent years (see, e.g., [5]).

Below, to demonstrate the richness of information contained in NCBI gene summaries, we examine the gene IFNA1 as an example:

> *Gene Symbol IFNA1 This gene is a member of the alpha interferon gene cluster on chromosome 9. The encoded cytokine is a member of the type I interferon family that is produced in response to viral infection as a key part of the innate immune response with potent antiviral, antiproliferative and immunomodulatory properties. This cytokine, like other type I interferons, binds a plasma membrane receptor made of IFNAR1 and IFNAR2 that is ubiquitously expressed, and thus is able to act on virtually all body cells. This cytokine is upregulated in preeclamptic placentas and is thought to be a mediator of preeclampsia.*

This summary begins by identifying IFNA1 as a member of the alpha interferon gene cluster on chromosome 9, immediately situating it within a specific gene family that plays a central role in the innate immune response. The summary further explains that the encoded cytokine is a type I interferon—a group known for its antiviral, antiproliferative, and immunomodulatory functions. Such details entail that IFNA1 is produced in response to viral infections, and its activity is critical for initiating an effective antiviral defense. Importantly, the summary also notes that IFNA1 binds to a receptor composed of IFNAR1 and IFNAR2, which are ubiquitously expressed on virtually all cell types. This implies that the cytokine's effect is systemic rather than limited to a specific tissue, underlining its fundamental role in immune regulation. Additionally, the summary mentions that this interferon is upregulated in preeclamptic placentas, hinting at a possible involvement in the pathophysiology of preeclampsia. Each of these elements—from gene cluster identity and receptor interaction to clinical associations—provides discrete, biologically relevant signals that a language model can capture in an embedding. The embedding of IFNA1 would thus reflect its association with antiviral defense, cytokine signaling, and immunomodulatory processes, which are crucial for drawing meaningful parallels with other genes engaged in similar biological functions.

To further validate that our approach—using an LLM to generate embeddings from gene descriptions—truly captures this rich information, we examined whether the high-dimensional representations reflect known biological relationships. If the embeddings effectively distill the semantic and functional nuances embedded in the gene descriptions, then genes with shared roles or pathways should naturally cluster together in the embedding space.

To test this, we selected a set of genes grouped by their known gene families and associated signaling pathways (see Table 1), and applied UMAP to project them into a two-dimensional space. The resulting UMAP plot (Figure 1) clearly shows that genes belonging to the same family form distinct clusters. Moreover, gene families within the same pathway cluster more closely than those from unrelated pathways, suggesting that the textual summaries encapsulate enough semantic information to differentiate among various signaling pathways and functional roles.

Notably, the interferon clusters (IFNA and IFNL) are positioned in close proximity, which aligns with the well-established fact that type I interferons share overlapping roles in innate immunity and signal transduction [6]. Similarly, the clusters representing hexokinases (HK) and phosphofructokinases (PFK) are closely apposed; both families are critical components of the glycolytic pathway, where HK catalyzes the first step of glucose phosphorylation and PFK serves as a key rate-limiting enzyme, underscoring their coordinated metabolic functions [7]. The RAS and MAPK clusters are also nearly overlapping, reflecting the intimate functional and regulatory connections within the Ras/MAPK signaling cascade that orchestrates cell proliferation, differentiation, and survival [8]. In addition, the proximity observed between the WNT2 and FZD clusters is particularly intriguing since WNT ligands interact with Frizzled receptors to activate the canonical and non-canonical Wnt signaling pathways, a relationship that is central to embryonic development and tissue homeostasis [9]. Furthermore, the clustering of PIK3 genes with PTEN demonstrates an expected biological balance [10, 11]; while PIK3 family members encode catalytic subunits that promote phosphoinositide 3-kinase activity, PTEN functions as a critical tumor suppressor by antagonizing this pathway. The spatial closeness of these clusters highlights the tight regulatory interplay governing cell growth and survival. Collectively, these observations substantiate the argument that embeddings generated from NCBI summaries encapsulate rich biological information.

It is also worth noting that the UMAP plot shown in Figure 1, which reduces the dimension of the embeddings from 1,536 to 2, losing a large proportion of information contained in the full-1,536-length embeddings; the full-length embeddings contain even much richer and detailed information.

3 Scouter accurately predicts types of genetic interactions

GEARS demonstrates impressive capabilities in predicting types of genetic interactions [2]. In this section, we evaluate Scouter's performance in this domain and compare it to that of

GEARS.

In the case of combinatorial gene perturbations, a naive approach might estimate the combined transcriptional response by simply adding the individual effects of each gene perturbation. However, biological systems frequently exhibit non-additive interactions, where the simultaneous perturbation of two genes leads to responses that are markedly different from the sum of their separate effects. For example, even when single-gene perturbations induce only minor changes in gene expression, the concurrent perturbation of two genes can trigger synergistic, suppressive, or even novel regulatory responses. To explore these complex dynamics, we evaluated Scouter's ability to predict various genetic interaction subtypes—such as synergy, suppression, redundancy, neomorphism, and epistasis—and directly compared its performance with that of GEARS. The following section presents our experimental design and findings, demonstrating that Scouter not only captures additive effects but also accurately predicts the nuanced non-linear interactions underlying combinatorial perturbations.

The classification of gene interactions is crucial for understanding how multiple gene perturbations influence cellular behavior. Building on the work from the Norman[12] dataset and GEARS[2], five primary non-additive GI subtypes have been defined: synergy, suppression, neomorphism, redundancy, and epistasis. For each GI type, they identified a corresponding list of two-gene perturbations (See Table 2), which serves as a benchmark for model evaluation. These classifications are based on experimentally derived GI scores that quantify the deviation from an additive effect.

Mathematical Framework for GI Scores Let $\bar{X}^{(a)} \in \mathbb{R}^d$ denote the average gene expression on d measured genes following perturbation on gene a, where $a = 0$ denotes the control state without any perturbation. Thus the average expression change induced by perturbation a relative to control state is given as:

$$\Delta^{(a)} = \bar{X}^{(a)} - \bar{X}^{(0)} \tag{2}$$

Given a two-gene perturbation $(a + b)$, a linear model is fitted:

$$\Delta^{(a+b)} = \beta_a \Delta^{(a)} + \beta_b \Delta^{(b)} + \epsilon \tag{3}$$

and solved with a Theil-Sen estimator (fit on 10,000 random subsamples of 1,000 genes at a time). With the estimated coefficients from the linear model, the type-specific GI scores are defined as in Table 3, including metrics like magnitude (computed as $\sqrt{\beta_a^2 + \beta_b^2}$), simi-

larity (via distance correlation), model fit, and equality of contribution. In our study, when predicting the GI score for a two-gene perturbation $(a+b)$, we use the predicted value $\hat{\Delta}^{(a+b)} = \hat{X}^{(a+b)} - X^{(0)}$ for equation 3, while taking the individual perturbation effects $\Delta^{(a)}$ and $\Delta^{(b)}$ directly from the ground truth data. This approach is slightly different from GEARS, which uses model-predicted values of $\Delta^{(a)}$ and $\Delta^{(b)}$, even though the individual perturbations a and b are directly included in the training data and thus their true values are known. Our method reflects a practical scenario—namely, when experimental data for single-gene perturbations are available, the real question is what happens when both genes are perturbed simultaneously. It avoids introducing additional uncertainty that could confound the detection/classification of the two-gene interaction effect.

Experimental Setup To rigorously evaluate Scouter's performance, we split the 72 curated gene perturbations in Table 2 into six distinct sets (each containing 12 perturbations). For each split, a separate Scouter model is trained, holding out one set as the test set and using all the other perturbations as the training set. After training, we compute the predicted GI scores for the test perturbations based on the definition in Table 3 using our defined framework, and repeat the same procedure for GEARS. This parallel setup allows for a direct, fair comparison between the two methods in both regression (accuracy of GI score prediction) and classification (ability to correctly label the GI subtype).

Regression Performance We evaluated Scouter and GEARS on their ability to predict four distinct GI scores—magnitude, model fit, equality of contribution, and similarity of transcriptional profiles—across all 72 two-gene perturbations (Figure 6). Each subplot compares predicted versus ground truth scores, with the first row corresponding to Scouter's predictions and the second row to GEARS's. The diagonal line $y = x$ serves as a visual reference for perfect agreement between predictions and ground truth. Overall, Scouter's points lie much closer to the diagonal, indicating more accurate GI score predictions.

To quantitatively measure the agreement between predictions and ground truth, we use mean squared error (MSE) and mean absolute error (MAE), which are displayed in the top-left corner of each subplot. It is clear that Scouter achieves lower MSE and MAE across all four GI scores compared to GEARS. On average, Scouter achieves a 53% decrease in MSE and a 43% decrease in MAE relative to GEARS. These findings confirm that Scouter is not only adept at modeling the overall transcriptional response to combinatorial perturbations but also excels at quantifying non-additive interactions, thereby providing a more nuanced and reliable framework for understanding complex gene regulatory mechanisms.

Classification Performance In addition to evaluating regression metrics for GI score prediction, we assessed Scouter's ability to classify each two-gene perturbation into its correct GI subtype. Treating each subtype as a one-versus-all classification problem, we computed the Area Under the Receiver Operating Characteristic Curve (AUC) using the relevant GI score. For instance, magnitude is used for synergy (higher values indicate stronger synergy), whereas equality of contribution is used for epistasis (lower values, once inverted, signify stronger epistasis). To address directionality issues in metrics where lower values imply a stronger interaction (such as epistasis), we inverted those scores before calculating the AUC.

Figure 7 displays the resulting ROC curves for each GI subtype, as well as a bar chart comparing the final AUC values for GI scores derived from Scouter and GEARS. Scouter exhibits consistently strong classification performance across all subtypes. In three out of the five subtypes, the performance difference between Scouter and GEARS is marginal—about 0.01 (suppression 0.80 vs. 0.79, neomorphism 0.66 vs. 0.67, and epistasis 0.85 vs. 0.86). However, Scouter significantly outperforms GEARS in redundancy (AUC of 0.91 for Scouter vs. 0.71 for GEARS). In synergy, Scouter achieves an AUC of 0.93, marking approximately a 3.3% improvement over GEARS.

Overall, these classification results confirm the findings from the regression metrics, illustrating that Scouter not only predicts GI scores accurately but also translates these scores into more precise subtype categorizations. By providing high AUCs for biologically important interactions such as synergy and redundancy, and maintaining competitive performance in the more complex epistasis subtype, Scouter offers a comprehensive and reliable framework for classifying combinatorial gene perturbations.

Recommendation Performance Beyond regression and classification, a critical real-world application of predictive models is their ability to recommend the most promising gene pairs for experimental validation. To evaluate this aspect, we measured Precision@10, defined as the fraction of the top 10 predicted interactions that truly exhibit a specific GI subtype. Figure 8 illustrates the results for both Scouter and GEARS across five GI subtypes.

Scouter and GEARS both achieve a Precision@10 of 0.9 for synergy, reflecting the models' strong performance in identifying highly synergistic gene pairs that deviate substantially from additive expectations. Among the remaining four GI subtypes, Scouter outperforms GEARS in three: 0.6 vs. 0.5 for suppression, 0.5 vs. 0.3 for epistasis, and 0.5 vs. 0.4 for redundancy. The only subtype in which Scouter underperforms compared to GEARS is neomorphism (0.4 vs. 0.5).

Taken together, these Precision@10 results confirm Scouter's strong capacity to prioritize functionally significant genetic interactions that merit experimental follow-up. In particular, its improvements in suppression, epistasis, and redundancy highlight Scouter's robustness in recognizing varied patterns of non-additive gene effects. This high-level recommendation capability is particularly valuable in laboratory settings, where researchers typically focus their resources on a limited set of top-ranked perturbations for in-depth validation.

Visual Illustrations To demonstrate Scouter's performance on specific two-gene perturbations, we selected three representative examples—one each for synergy, suppression, and epistasis—and plotted their respective differentially expressed genes in Figure 9. In each subplot, the hatched bars show the effects of perturbing gene a (striped) and gene b (dotted) independently. Two individual perturbations are stacked if they share the same direction of change. The colored bars represent the predicted outcomes of the two-gene perturbation from Scouter (orange) and GEARS (pink), while the gray bars reflect the true observed changes.

In the synergy example (top panel of Figure 9), the true combined effect (gray bar) is substantially greater than the sum of the individual perturbations (hatched bars), signifying a strong non-additive interaction. Scouter's predictions closely mirror these amplified expression changes across multiple genes, effectively capturing the hallmark of synergy: a combined outcome that exceeds what would be expected from simple addition. By contrast, GEARS systematically underestimates the magnitude of this interaction, resulting in smaller changes than those observed in the ground truth. This underestimation is especially evident in genes like HBG2 and BLVRB, which even indicates a suppressive prediction.

For suppression (middle panel of Figure 9), the true combined effect is more subdued than the sum of the individual gene perturbations. While each gene alone (hatched bars) induces a pronounced increase or decrease in expression, the double perturbation (gray bars) partially cancels out these individual effects. Scouter again demonstrates stronger alignment with the observed suppression pattern, particularly in genes like AIF1 and SH3BGRL3. GEARS, on the other hand, tends to misjudge the extent of suppression in several genes, occasionally predicting changes in the opposite direction. These discrepancies highlight Scouter's enhanced sensitivity to interactions where one perturbation dampens or blunts the other.

Epistasis arises when one perturbation's effect masks or overshadows the other's. In the bottom panel of Figure 9, the true combined outcome (gray bars) largely reflects the dominant effect of one perturbation, with certain genes (e.g., GPSM3, S100A11) showing

expression changes closer to what would result from perturbing MAPK1 alone. Scouter's predictions more accurately follow these epistatic patterns, particularly in capturing the direction and approximate magnitude of change in genes such as TMSB4X and LIMD2. While GEARS also detects a masking trend, it consistently underestimates the absolute magnitude of the combined perturbation in several genes (e.g., GAL, AC079466.1), underscoring Scouter's superior ability to model the nuanced interplay of epistasis.

Taken together, these three examples illustrate how Scouter excels at modeling diverse non-additive interactions. Whether the outcome is synergistic amplification, suppressive dampening, or epistatic dominance, Scouter's predicted gene expression profiles closely track empirical measurements, thereby offering a more reliable and biologically informative picture of two-gene perturbations.

We did not include biolord in this section, because biolord manually adds the effect of each single-gene perturbation as its prediction for the multi-gene perturbation:

$$\Delta^{(a+b)} = \Delta^{(a)} + \Delta^{(b)} \tag{4}$$

This approach always renders a perfect additive effect and fails to capture non-additive interactions.

4 Ablation study: the performance of Scouter with degraded LLM-based embeddings

In the main text, we have demonstrated the superior performance of Scouter, which utilizes highly informative LLM-based embeddings as inputs. To assess the critical role of these LLM-based embeddings in Scouter's performance, we conducted an ablation study. In this study, we intentionally degraded the NCBI gene descriptions in three different ways, generated text embeddings from these altered descriptions, and then evaluated the impact on Scouter's performance.

Picking the first $K\%$ of words from the NCBI summary text In this experiment, starting with only gene names, we progressively increase the percentage of words retained, and pass them to the text embedding model to generate the embeddings. Specifically,

- K = 10: Only genes names are kept, e.g., "Gene: APOE"
- K = 20: First 20% words of the full NCBI gene summary are kept.

- ...
- K = 100: The original NCBI gene description without any deletion.

For $K = 10$, we intentionally define the truncated text to include only the gene name (e.g., "Gene: APOE") to represent an extreme case of minimal retention, although the literal first 10% of the full NCBI summary would typically be longer.

Once the embeddings from the truncated versions of gene summaries are generated, the subsequent steps remain the same. The results, presented in Figure 2j of the main text, reveal that as we progressively reduce the textual information provided in the gene descriptions—from full details down to just the gene name—the prediction accuracy is gradually compromised. The normalized MSEs with $K = 10$ on the Dixit, Adamson, K562, and RPE1 datasets are 117%, 99%, 30%, and 8% higher, respectively, than those with $K = 100$. However, the normalized MSEs under any K value are still less than 1, indicating that Scouter, based on weakened embeddings, still performs better than the baseline. (The normalized MSEs under $K = 100$ may slightly differ from those in Figure 2 of the main text. This discrepancy arises because Figure 2 utilizes embeddings directly downloaded from the GenePT paper, whereas in this ablation study, we derive the NCBI gene summaries and obtain the OpenAI embeddings independently. Variations in the extraction of NCBI summaries (e.g., differences in whitespace, lowercase, or newline characters), updates to OpenAI's embedding model over time, and other minor preprocessing differences can contribute to these variations.)

It's worth noting that even when only the gene name was used to generate embeddings (i.e., $K = 10$), the prediction accuracy of Scouter remained acceptable. We interpret this result as evidence that the LLM we used has internalized a vast amount of background biological knowledge during its pre-training, and thus it is still able to generate highly informative embeddings even without additional description of the gene. Take "APOE" as an example: the model does not simply read it as a string of random characters; rather, it recognizes "APOE" as a gene and automatically retrieves a rich repository of knowledge it gained from pre-training, including the gene's functions, interactions, and relevance in various biological contexts. See an example conversation in Figure 3 with OpenAI's ChatGPT-4 where we only input "Gene APOE"; the model is still able to render the correct biological function description about the provided gene. This intrinsic understanding allows the model to produce acceptable embeddings even with minimal additional input. While detailed gene descriptions do enhance performance, our findings suggest that the LLM's pre-learned biological context provides a strong foundation for extrapolating gene perturbation outcomes—a resilience

that is particularly promising for applications where comprehensive gene annotations may not be available.

Randomly picking $K\%$ of words from the NCBI summary text This ablation study is similar to the previous study in terms of reducing information. We randomly pick $K\%$ of the words from the summary text, resulting in a selected text with a scrambled description. The truncation of information in this study is more drastic than in the previous one: in addition to the loss of words, the text structure is also disturbed in this case. For example, the full NCBI summary of gene PTEN is as below:

> *This gene was identified as a tumor suppressor that is mutated in a large number of cancers at high frequency. The protein encoded by this gene is a phosphatidylinositol-3,4,5-trisphosphate 3-phosphatase. It contains a tensin like domain as well as a catalytic domain similar to that of the dual specificity protein tyrosine phosphatases. Unlike most of the protein tyrosine phosphatases, this protein preferentially dephosphorylates phosphoinositide substrates. It negatively regulates intracellular levels of phosphatidylinositol-3,4,5-trisphosphate in cells and functions as a tumor suppressor by negatively regulating AKT/PKB signaling pathway. The use of a non-canonical (CUG) upstream initiation site produces a longer isoform that initiates translation with a leucine, and is thought to be preferentially associated with the mitochondrial inner membrane. This longer isoform may help regulate energy metabolism in the mitochondria. A pseudogene of this gene is found on chromosome 9. Alternative splicing and the use of multiple translation start codons results in multiple transcript variants encoding different isoforms.*

In the case of $K = 10$, the text provided to the text embedding model might become:

> *of start Alternative suppressor be domain is this at intracellular isoforms. tumor transcript the preferentially as*

We tested $K = 10, 20, \cdots, 100$ and present the results in Figure 4. Overall, we observe a more clear decrease in prediction accuracy compared to the previous study. On average, the normalized MSE with $K = 10$ is 72% higher than that with $K = 100$. This aligns with the expectation that with an additional decrease in information beyond just truncation, the embeddings increasingly struggle to capture the essential biological information of genes.

Scrambling gene descriptions before computing GenePT embeddings Here we consider scrambling the gene descriptions by shuffling the words of the original NCBI text

before they are provided to the text embedding model. It represents a special case of a previous ablation study when $K = 100$, as randomly selecting all words without replacement essentially scrambles the text. We have drawn dotted lines in Figure 4 to indicate the performance of Scouter under $K = 100$ without sampling or shuffling of words. On average, the scrambled version of the embedding results in a 25% higher normalized MSE than the ordered version, indicating that the full context provided by ordered text does enhance performance.

It's also worth noting that the performance gap is not drastic. This finding suggests that even when the semantic structure is perturbed, the LLM is still able to extract meaningful biological information—likely because it has internalized extensive background knowledge during pre-training. In other words, the model recognizes key elements such as gene names and critical biological terms regardless of their order, though preserving the original word order does confer a measurable advantage by maintaining the full contextual richness. We illustrate the capture of information even with scrambled text via a conversation with ChatGPT-4o, as shown in Figure 5. Even though we provided a scrambled text of the PTEN gene's NCBI summary, without even mentioning the gene "PTEN," the model still accurately identified that the description pertains to the PTEN gene.

5 Using LLM-based embeddings for GEARS and biolord

It is intriguing to explore whether introducing gene embeddings derived from LLMs can enhance existing predictive models, and so we conduct the relevant experiments. In the GEARS model, GO terms are used to construct a perturbation graph where each perturbation has a node embedding, which is initialized randomly and updated during training to optimize the loss function. We modified the code to replace these perturbation embeddings with gene embeddings derived from LLMs and freeze them throughout the training process. In biolord, each perturbation is treated as an ordered attribute, represented by a GO terms-derived vector as shown in equation 1, which is not updated. We similarly replaced this vector in biolord with gene embeddings derived from LLMs.

We conducted the experiments on all four single-gene datasets, and the result is given in Figure 2k of the main text. Overall, replacing original embedding derived from GO-terms with embeddings from LLM introduces improvement on prediction accuracy for both biolord and GEARS on most datasets. biolord still fails on Dixit dataset, obtaining normalized MSE 21.50 and 21.58 with LLM embedding and GO-term derived embeddings, respectively. On average, compared with GO term-derived embeddings, GEARS with LLM embeddings de-

creases the normalized MSE by 14% (median 5%), and biolord lowers by 2% (median 3%). The performance improvement of GEARS and biolord is evident but modest.

The above results suggest that Scouter's significantly better performance compared to GEARS and biolord is attributed to both the use of LLM-based embeddings, which contain much richer information about genes and their relationships, and the neural network architecture that efficiently leverages this rich information for predicting transcriptional responses.

6 Comparison with gene-expression foundation models

Before presenting the benchmarking results, we would like to briefly highlight several advantages of Scouter relative to gene-expression foundation models—advantages that go beyond predictive accuracy and speak to accessibility and ease of deployment.

First, like GEARS and biolord, Scouter is a task-specific model designed explicitly for predicting transcriptional responses to genetic perturbations. Unlike general-purpose foundation models that aim to learn broad representations across diverse scRNA-seq datasets, Scouter is built for a focused, well-defined task. This specialization allows for a simpler and more efficient architecture: a compressor–generator framework that disentangles cell state and perturbation input, and integrates LLM-based gene embeddings in a lightweight, fixed manner—without requiring fine-tuning or retraining the embedding model.

Second, Scouter requires no pre-training. It can be trained end-to-end on a single Perturb-seq dataset using minimal computational resources—a single modest GPU (e.g., an A40 or lower) or even a CPU (e.g., the Apple M2 chip in a MacBook Pro)—and typically completes training in under an hour. In contrast, models like scFoundation require extensive pre-training on large-scale scRNA-seq collections, high-end hardware (e.g., A100 80 GB GPUs), and substantial engineering effort.

Third, Scouter may generalize better to perturbed or diseased conditions. Foundation models are typically pre-trained on datasets composed primarily of healthy, unperturbed cells, which can lead to distributional mismatch and degraded performance when applied to pathological or experimentally perturbed states. In contrast, Scouter leverages gene embeddings from general-purpose LLMs (e.g., GPT), which are trained on diverse text corpora. These embeddings are derived from NCBI gene descriptions, which often capture gene function across a broad spectrum of biological contexts, including disease.

Taken together, these advantages highlight Scouter's accessibility, specificity, and practicality. With that context in mind, we now present the benchmarking results comparing

Scouter to scFoundation and two other recent gene-expression foundation models: scGPT [13] and scELMo [14]. We conducted benchmarking experiments against these three models using the Adamson and Norman datasets, which were also used as benchmark datasets in the original papers of these models. For each method, we followed the official reproduction scripts provided in their public repositories. The only modification we made was to run five different random seeds (1 through 5) for data splitting, instead of relying on a single fixed split. In each seed, we held out 20% of perturbations for testing and used the remaining 80% for training. By reporting median results across five repetitions, we account for variability due to random partitioning and ensure a fairer and more reproducible comparison.

On the Adamson dataset, we first compared Scouter against scGPT and scELMo using normalized MSE and 1-PCC. Since both models benchmark against GEARS in their original papers, we also re-plotted GEARS's performance alongside scELMo and scGPT to demonstrate that our reproduction pipeline faithfully replicates the reported results. As shown in Figure 2l in the main text, GEARS achieves a median normalized MSE of approximately 0.186 and a median 1-PCC of 0.050. These values are only slightly improved by scELMo, which reaches about 0.151 and 0.040, closely matching the trend in Extended Data Figure 13a of the scELMo paper. scGPT achieves comparable performance, with a normalized MSE of 0.162 and 1-PCC of 0.036. Nevertheless, Scouter's performance remains clearly superior: using a compressor–generator architecture fed with LLM-derived gene embeddings, Scouter achieves a normalized MSE of only 0.093 and a 1-PCC of approximately 0.021—representing an additional 43% reduction in MSE and 42% in 1-PCC relative to scGPT. Overall, Scouter reduces normalized MSE by about 50% and 1-PCC by approximately 58% compared to the original GEARS baseline. These results underscore the effectiveness of combining LLM-based gene embeddings with a lightweight, task-specific neural network.

We next extended our analysis to the Norman dataset, which includes both single- and two-gene perturbations. Following established convention, we divided two-gene perturbations into three categories: "seen0" (neither gene observed individually during training), "seen1" (only one gene observed), and "seen2" (both genes observed separately). Single-gene perturbations were treated as a fourth category. The results are shown in Figure 2m in the main text. In the seen0 case, GEARS yields a median normalized MSE of about 0.434, while scELMo and scGPT perform slightly worse at around 0.496 and 0.634, respectively. In contrast, Scouter reduces the error to about 0.083—a 81% improvement relative to GEARS. In the seen1 scenario, GEARS scores about 0.345, scELMo 0.346, and scGPT 0.470, whereas Scouter achieves 0.069, representing an 80% improvement over the best of the other meth-

ods. Even in the seen2 case, which is the easiest among the two-gene categories, GEARS, scELMo, and scGPT cluster between 0.221 and 0.318, while Scouter reaches just 0.054, lowering error by about 76% relative to the next best model. For single-gene perturbations, Scouter again outperforms all competitors, achieving a normalized MSE of 0.164, compared to 0.687 for GEARS, 0.667 for scELMo, and 0.787 for scGPT—reducing error by over 76%. The 1 – PCC metric, shown in Figure 2n in the main text, reveals a consistent pattern: in the seen0 case, Scouter scores around 0.038, compared to 0.133 for GEARS and higher values for the other two models. In seen1 and seen2, Scouter achieves 0.019 and 0.014, respectively, far below the 0.065–0.120 range for the other methods. Even for single-gene predictions, Scouter's 1 – PCC of 0.072 is well below the 0.199–0.378 range seen in other models.

It is worth noting that our results on the Norman dataset differ slightly from those reported in the original scGPT and scELMo papers, which claimed marginal improvements over GEARS. However, their reported gains were modest—less than 5%—and likely reflect differences in data splits, seeds, or evaluation methodology. Based on our inspection of their released training scripts, it appears their results are based on a single train-test split, whereas our results are averaged across five random splits for greater stability. Our evaluation is also more comprehensive: the scGPT paper reports only PCC, not normalized MSE; both papers report aggregate results across all perturbations without distinguishing between seen0, seen1, seen2 and single categories. In contrast, we report both MSE and PCC across these biologically meaningful subgroups, following conventions established by GEARS and biolord. We believe this more granular analysis provides a clearer and more informative assessment of model performance.

Taken together, our results show that Scouter consistently outperforms scGPT and scELMo by a wide margin, and this conclusion holds regardless of whether one uses our evaluation or the results reported in the original foundation model papers.

We now turn to our comparison with scFoundation, highlighting the practical challenges we encountered, the workaround we adopted, and the resulting performance differences that further underscore Scouter's advantages.

Our initial attempts to fine-tune scFoundation for perturbation prediction ran into a fundamental limitation: GPU memory exhaustion. scFoundation tokenizes each single-cell expression profile as a "sentence" of approximately 20,000 gene tokens. Due to the quadratic memory complexity of self-attention, each attention head must store on the order of 370 million float values—roughly 1.5 GB—just for the attention score matrix. Despite using an NVIDIA A40 with 48 GB of memory, the model failed immediately upon allocating the

first attention tensor. Other users reported similar issues in scFoundation's GitHub repository (`https://github.com/biomap-research/scFoundation`, see issues #33 and #68), and the authors themselves recommend using an A100 80 GB GPU even for inference. According to issue #68, fine-tuning the model for perturbation prediction takes approximately 55 hours on an A100 80 GB GPU, even when transformer weights are frozen. From a practical standpoint, requiring such high-end hardware and multi-day runtimes to adapt a pre-trained model just to a regression task involving a single dataset places it out of reach for many academic labs and applied research groups.

Nevertheless, we felt it was important to include scFoundation in our benchmarking. To do so transparently and fairly, we adopted the metric values reported in the original scFoundation paper (Figure 5b), which presents raw MSE (not normalized MSE; see line 91 of their inference code `https://github.com/biomap-research/scFoundation/blob/main/GEARS/gears/inference.py#L91`). We then computed raw MSE for Scouter and GEARS using the same convention, applying it to the Norman dataset. The results are presented in Figure 2o in the main text. For single-gene perturbations, Scouter achieves an average MSE of 0.095, compared to 0.188 for scFoundation and 0.234 for GEARS. In the most difficult seen0 category, Scouter reaches 0.054, less than a quarter of scFoundation's 0.230 and only 13% of GEARS's 0.401. Even in the intermediate seen1 and seen2 categories, Scouter's MSE values of 0.055 and 0.040 outperform scFoundation's 0.184 and 0.110, as well as GEARS's 0.244 and 0.149, respectively.

It is important to note that our results differ somewhat from scFoundation's Figure 5b. For instance, they report an MSE of around 0.24 for GEARS on the seen0 category, while we observe a higher MSE of 0.401. This discrepancy likely stems from differences in evaluation protocol: scFoundation's numbers are based on a single fixed train-test split (seed = 1), as shown in their reproduce notebook `https://github.com/biomap-research/scFoundation/blob/main/GEARS/Plot.ipynb`, whereas we report median results over five random train-test splits. We believe this five-fold repetition provides a more robust and reproducible estimate of model performance. It is also possible that certain differences arise indirectly from computational resource constraints, which limited our ability to replicate scFoundation's full training protocol.

The benchmarking against scFoundation ultimately highlights two key points. First, even if one generously credits scFoundation with the best-case results reported in its paper, Scouter still achieves substantially lower error—despite being trained in under seven minutes on a single A40 GPU. Second, scFoundation's need for 80 GB of VRAM and multi-day fine-tuning poses a major accessibility barrier. In contrast, Scouter offers a lightweight,

highly efficient alternative that democratizes access to high-quality perturbation prediction. These findings underscore that Scouter's streamlined compressor–generator architecture, powered by LLM-derived gene embeddings, delivers not only markedly superior accuracy but also a dramatic reduction in computational cost.

7 Exploring cell-type-specific gene embeddings

It is well known that the function of a gene—and, more broadly, the structure of gene-regulatory networks—can vary substantially across cell types. This suggests that gene embeddings that reflect such context-specific regulatory roles could improve model performance, both for within-dataset prediction and for generalization across cell types. If such differences were captured in natural language descriptions and included in the input to an LLM, then the resulting embeddings might better reflect cell-type-specific regulatory roles and thereby yield more accurate predictions of transcriptional responses.

In practice, however, implementing this idea is challenging. While it is widely accepted that gene regulation is highly context-dependent, the precise differences in gene function across cell types are often not fully understood—and, more importantly for this task, are rarely described in accessible textual form. As a result, cell-type-specific regulatory information is typically not available in a format that can be directly incorporated into an LLM embedding pipeline.

Nonetheless, to explore this idea in a first approximation, we conducted a proof-of-concept experiment in which we generated naively cell-type-specific gene embeddings by appending a brief description of the relevant cell type to the canonical NCBI gene description. Specifically, for each gene–cell-type pair (e.g., gene TP73 in the K-562 cell line), we concatenated the gene's NCBI entry with the cell line description from the American Type Culture Collection (ATCC). For example, the text provided to the LLM for generating the TP73–K-562 embedding was:

> *Gene Symbol TP73 This gene encodes a member of the p53 family of transcription factors involved in cellular responses to stress and development. It maps to a region on chromosome 1p36 that is frequently deleted in neuroblastoma and other tumors, and thought to contain multiple tumor suppressor genes. The demonstration that this gene is monoallelically expressed (likely from the maternal allele), supports the notion that it is a candidate gene for neuroblastoma. Many transcript variants resulting from alternative splicing and/or use of alternate promoters have been found for this gene, but the biological validity and the full-length nature of some variants*

have not been determined. K-562 are lymphoblast cells isolated from the bone marrow of a 53-year-old chronic myelogenous leukemia patient. The K-562 cell line is widely used in immune system disorder and immunology research. The K-562 cell line has attained widespread use as a highly sensitive in vitro target for the natural killer assay. Cultures from the ATCC stock have been shown to exhibit this sensitivity for assessing human natural killer activity.

Here, the black text represents the NCBI description of gene TP73, while the royal blue text is the ATCC cell line description for K-562. This combined text was then supplied to the OpenAI text-embedding-ada-002 model to produce a cell-type-specific gene embedding.

Figure 16 illustrates the difference between the original gene embeddings—based solely on NCBI descriptions—and the cell-type-specific embeddings generated using the naive method described above. Each point in the plot represents a gene perturbed in one or more of the Dixit, Adamson, and Norman datasets. Notably, all three datasets were generated using the K-562 cell line, so the same ATCC cell-type description was appended for all genes. Lines connect the two embeddings (original vs. cell-type-specific) for each gene, showing how the embedding shifts when the cell-type description is added. For visual clarity, we display lines for only ten randomly selected genes, though the pattern holds broadly across the dataset.

As the figure shows, the direction and magnitude of the shift are highly consistent across all genes. This suggests that, to a first approximation, the transformation from original to cell-type-specific embeddings can be described as:

$$B_{\text{cell-type-specific}}(g) = B_{\text{original}}(g) + C,$$

where B denotes the embedding of gene g, and C is a constant vector that is independent of the specific gene.

In the context of within-dataset prediction—Scouter's primary setting—such a global, input-invariant shift is unlikely to improve model performance. Since all gene embeddings are shifted by approximately the same vector C, this difference can be absorbed by the bias term in the first layer of the neural network and does not meaningfully change the relative geometry of the embedding space.

To test this hypothesis empirically, we conducted an experiment on the Adamson dataset, which contains K-562 cells. We repeated the full training and evaluation pipeline described in the main text, comparing two embedding strategies: (1) embeddings derived from NCBI gene descriptions alone, and (2) embeddings derived from the same descriptions appended

with the K-562 cell-line annotation. The results are shown in Figure 17, which reports the median normalized MSE and 1–PCC under both settings. As expected, the two embedding strategies yielded nearly identical predictive performance across both metrics. These results support our theoretical rationale: if the added cell-type annotation induces only a dataset-wide constant shift in the embedding space, it does not improve the discriminative power of the inputs and thus offers no tangible benefit for within-dataset prediction.

8 Scouter's performance on cross-dataset prediction

The current version of Scouter is designed to be trained individually on each Perturb-seq dataset. Its primary goal is to extrapolate transcriptional outcomes within a given dataset: due to technical constraints, most Perturb-seq experiments perturb only a relatively small number of genes or gene pairs. Scouter leverages the data from these experimentally perturbed conditions to predict the transcriptional effects of additional, unperturbed genes or gene combinations—always within the same biological condition or cell type. In this regard, Scouter shares the same problem formulation and scope as GEARS and biolord, which also operate within a dataset-specific, condition-matched framework. None of these methods, including Scouter, were originally developed to support cross-dataset or cross-cell-type predictions.

Is it possible to training a Scouter model on one Perturb-seq dataset and using it to predict perturbation outcomes in a different dataset, such as a different cell type or condition? While Scouter was not designed for this purpose, we were eager to explore whether it might show some promise. To this end, we conducted a set of pilot cross-condition experiments involving Scouter, GEARS, and biolord. All models were trained exclusively on the Adamson dataset, which contains CRISPRi-based gene perturbations in K562 cells. We then evaluated their performance on two test datasets: (1) Replogle K562, which also involves K562 cells (but there are differences in the K562 cells used in these two studies: In the Adamson dataset, K562 cells were engineered to express varying levels of dCas9-KRAB, including a high-expression variant (cMJ006), to optimize multiplexed CRISPRi perturbations. In contrast, the Replogle K562 dataset used a pre-validated monoclonal K562-dCas9-KRAB cell line for robust and scalable genome-wide Perturb-seq screening.) and thus represents a mild domain shift, and (2) Replogle RPE1, which involves a different cell line (RPE1) and represents a more substantial shift in both biology and technical conditions. Following the evaluation strategy used in our main results, we assessed performance on the top 20 differentially expressed genes (DEGs) using normalized MSE and Pearson correlation coefficient

(PCC).

As shown in Figure 15, Scouter outperformed both biolord and GEARS in both cross-dataset settings. However, all three models exhibited a substantial drop in accuracy compared to their within-dataset results. For the mild shift (Replogle K562), Scouter's normalized MSE rose to 2.48—about five times higher than its within-dataset error of $\sim$0.51—and its PCC dropped to 0.29 (vs. $\sim$0.75 within-dataset). Biolord and GEARS performed worse, with MSEs above 3.1 and PCCs below 0.29. Under the more severe shift (Replogle RPE1), performance degraded further: normalized MSEs increased to 2.72 (Scouter), 3.44 (biolord), and 3.67 (GEARS), while PCCs fell to 0.10–0.13 across all methods. Notably, all normalized MSEs exceeded 1, indicating that none of the models outperformed a naïve baseline that simply uses the average expression profile of control cells.

These findings, although discouraging, are biologically and technically unsurprising. Cross-condition prediction requires a model to transfer a causal mapping that is itself highly context-dependent—and decades of functional-genomics work show just how strongly that mapping varies:

1. Cell-type– and condition-specific regulatory circuitry. Gene-regulatory networks rewire across cell types, developmental stages, and disease contexts; consequently, the same CRISPRi perturbation can trigger different—or even opposite—transcriptional cascades in K562 leukemia cells versus RPE1 epithelial cells. Large-scale projects such as GTEx and ENCODE have documented thousands of tissue-specific eQTLs and enhancer–promoter connections, while perturb-seq screens reveal marked heterogeneity in gene-knockdown effects across lineages (e.g., [19, 12, 20]). Any model trained on one network is therefore liable to mis-specify causal edges in another context.

2. Divergent molecular baselines. Baseline transcription, chromatin accessibility, and signaling-pathway activity differ markedly across cell lines and environmental conditions [21, 22]. Because Scouter and its peers learn residual changes relative to those baselines, a shift in the starting state amplifies error in the predicted delta.

3. Batch and protocol heterogeneity. Even within the same cell type, labs differ in guide-RNA libraries, CRISPRi efficiency, library-prep chemistries, sequencing depth, and cell-cycle composition—all well-known sources of batch effects in single-cell data [23, 24]. These technical shifts compound biological ones and further degrade cross-dataset transfer.

In short, the sharp performance drop is a consequence of fundamental biological variabil-

ity plus unavoidable experimental heterogeneity—challenges that no existing perturbation-prediction model, including Scouter, has yet overcome.

9 Robustness of prediction to control cell sampling

To study the robustness of Scouter's prediction to the choice of K and the random seed, we conducted a comprehensive evaluation on the Adamson dataset. We created a two-dimensional testing grid by varying the number of randomly sampled control cells from 100 to 1000 in increments of 100, and by using ten different random seeds. This yielded 100 distinct configurations. For each configuration, we repeated the training and evaluation five times using different train–test split seeds and reported the median normalized MSE and 1 – PCC across the five runs. We then grouped the performance metrics by both the number of control cells and the random seed to assess variability.

The results are summarized in Figure 10. As shown, Scouter's performance remains highly stable across all tested settings. Both normalized MSE and 1 – PCC values show minimal variation with respect to either the number of control cells or the choice of random seed.

To further illustrate this point, we examined a specific case: prediction of the transcriptional response to ATP5B perturbation. We compared predictions from two highly different configurations—100 control cells with seed 10 versus 1000 control cells with seed 1. As shown in Figure 11, the predicted expression profiles for the top 20 DEGs are highly similar between these two settings.

In summary, these experiments demonstrate that Scouter's predictive performance is robust to the number of sampled control cells and the choice of random seed.

10 Additional baselines

In the main text, we have compared Scouter with two state-of-the-art methods, GEARS and biolord. Below, we will present two heuristic baseline approaches that do not utilize LLM embeddings. The results demonstrate that Scouter consistently outperforms all these baselines.

Notation: Let $X^{(g)} \in \mathbb{R}^{N_g \times d}$ be the gene expression matrix for cells following the perturbation of gene g, where N_g is the number of cells under perturbation g, and d is the number of genes measured. The case $g = 0$ denotes control condition without any perturbation. The

mean expression vector for a perturbation g is given by

$$\bar{X}^{(g)} = \frac{1}{N_g} \sum_{i=1}^{N_g} X_i^{(g)},$$

where $X_i^{(g)}$ is the expression vector of the ith cell under perturbation g.

Baseline 1: Control Average Prediction This baseline predicts that a new gene perturbation g^* causes no change from the control state:

$$\hat{X}^{(g^*)} = \bar{X}^{(0)}.$$

This baseline represents the simplest null hypothesis: if a perturbation has no effect, the control state serves as the best guess. This baseline model corresponds to a normalized MSE of 1. Therefore, if the normalized MSE of a predictive model is less than 1, then the model outperforms this baseline. As reported in the main text, Scouter achieves a normalized MSE of less than 1 across all datasets.

Baseline 2: Training Average Prediction A second baseline to predict the outcome of an unseen gene perturbation is to use the average effect observed across all known perturbations in the training data. Let $\mathcal{G}_{train}$ be the set of perturbed genes in the training data, which does not include the perturbation g^* of interest. Then, the prediction is given by:

$$\hat{X}^{(g^*)} = \frac{1}{|\mathcal{G}_{train}|} \sum_{g \in \mathcal{G}_{train}} \bar{X}^{(g)} \quad (5)$$

This baseline is motivated by the intuition that, in the absence of gene-specific embeddings, the overall trend in perturbation effects offers a reasonable guess. It aggregates the average response observed in conducted perturbation experiments, providing a pragmatic reference against which the performance gain from incorporating detailed gene embeddings—as done in Scouter—can be measured. We compared Scouter to such baseline to all the four one-gene datasets and give the result in Figure 2. It is worth noting that, except on the Dixit dataset, which has too few perturbations, the training average baseline actually achieves better performance than the control average, as evidenced by a normalized MSE of less than 1. Scouter consistently outperforms the training average across all datasets, with a normalized MSE on average 53% smaller than that of the training average.

11 Robustness of predictive accuracy across varying numbers of DEGs

We inherited the use of the top 20 differentially expressed genes (DEGs) for performance evaluation from the GEARS and biolord studies, which facilitates direct and fair comparison.

To explore the performance of Scouter across a broader range of DEGs, we conducted an extended analysis on the Adamson dataset. For each method—Scouter, biolord, and GEARS—we computed the median normalized MSE and median 1 – PCC across the top K DEGs, varying K from 20 to 100. The results, shown in Figure 12, indicate that Scouter consistently outperforms both biolord and GEARS at every cutoff. While all methods exhibit a slight increase in normalized MSE as K increases—from 0.100 to 0.122 for Scouter, 0.141 to 0.163 for biolord, and 0.186 to 0.217 for GEARS—the performance gap remains stable. Similar trends are observed in the 1 – PCC metric: Scouter's values increase from 0.023 to 0.049, compared to 0.028 to 0.052 for biolord and 0.050 to 0.085 for GEARS. These results confirm that Scouter's superior predictive accuracy is robust to the choice of DEG cutoff.

To better understand why performance degrades slightly as more DEGs are included, we conducted an exploratory analysis of perturbation magnitudes. As shown in Figure 13a, the median number of genes per perturbation with a log-normalized mean expression difference (perturbed vs. control) exceeding various thresholds (0.25 to 0.65) remains small, indicating that only a limited number of genes experience substantial expression changes. A companion histogram in Figure 13b shows that most non-zero expression values are under 2 units, with large shifts being relatively rare. Together, these findings suggest that focusing on the top 20 DEGs is a reasonable and effective strategy for capturing the core perturbation signal. Including more genes inevitably introduces less informative or weakly perturbed genes, which dilutes normalized performance metrics without offering additional biological insight.

12 Assessing distributional accuracy with energy distance

In the Perturb-seq literature, energy distance is commonly used to quantify the overall magnitude of a perturbation by comparing the empirical distribution of perturbed cells to that of control cells [15, 16, 17, 18]. Typically, researchers embed high-dimensional gene expression profiles into a lower-dimensional space, compute pairwise distances between control and perturbed samples, and summarize these distances as an effect-size measure to rank or cluster perturbations.

However, energy distance is not directly applicable for evaluating GEARS or biolord, as both methods output a single predicted expression vector per perturbation rather than a distribution of predicted cells. Because energy distance fundamentally quantifies separation between two distributions, it becomes unstable or degenerate when one distribution collapses to a single point. Consequently, applying energy distance to these methods would require altering their inference protocols in ways that deviate from their original published implementations.

In contrast, energy distance can be readily computed for Scouter, but a meaningful evaluation requires an appropriate baseline for comparison. To this end, we define a normalized energy-distance ratio:

$$\text{e-distance}(\hat{y}, x)/\text{e-distance}(y, x),$$

where x, y, and $\hat{y}$ represent the distributions of control cells, true perturbed cells, and Scouter-predicted perturbed cells, respectively. This ratio quantifies how closely the model's predicted global shift aligns with the true perturbation effect size; a value close to 1 indicates high fidelity.

To mitigate the effects of curse of high dimensionality, we computed this ratio in two types of reduced-dimensional spaces (see Figure 14 for results). First, we restricted the expression profiles to the top K differentially expressed genes (DEGs), varying K from 20 to 50. The resulting ratios were 1.05, 1.10, 1.14, and 1.18 for $K = 20$, 30, 40, and 50, respectively. Second, we projected the full expression profiles onto their first K principal components (PCs), again varying K from 20 to 50. This yielded ratios of 1.29, 1.31, 1.34, and 1.37, respectively. These modest deviations from 1 indicate that Scouter reliably captures the global magnitude of perturbation effects.

References

[1] Michael Ashburner, Catherine A Ball, Judith A Blake, David Botstein, Heather Butler, J Michael Cherry, Allan P Davis, Kara Dolinski, Selina S Dwight, Janan T Eppig, et al. Gene ontology: tool for the unification of biology. *Nature genetics*, 25(1):25–29, 2000.

[2] Yusuf Roohani, Kexin Huang, and Jure Leskovec. Predicting transcriptional outcomes of novel multigene perturbations with gears. *Nature Biotechnology*, 42(6):927–935, 2024.

[3] Zoe Piran, Niv Cohen, Yedid Hoshen, and Mor Nitzan. Disentanglement of single-cell data with biolord. *Nature Biotechnology*, 42(11):1678–1683, 2024.

[4] Dennis A Benson, Mark Cavanaugh, Karen Clark, Ilene Karsch-Mizrachi, David J Lipman, James Ostell, and Eric W Sayers. Genbank. *Nucleic acids research*, 41(D1):D36–D42, 2012.

[5] Chongyang Tao, Tao Shen, Shen Gao, Junshuo Zhang, Zhen Li, Zhengwei Tao, and Shuai Ma. Llms are also effective embedding models: An in-depth overview. *arXiv preprint arXiv:2412.12591*, 2024.

[6] Sidney Pestka, Christopher D Krause, and Mark R Walter. Interferons, interferon-like cytokines, and their receptors. *Immunological reviews*, 202(1):8–32, 2004.

[7] Jeremy M Berg, John L Tymoczko, and Lubert Stryer. *Biochemistry (loose-leaf)*. Macmillan, 2007.

[8] Julian Downward. Targeting ras signalling pathways in cancer therapy. *Nature reviews cancer*, 3(1):11–22, 2003.

[9] Hans Clevers and Roel Nusse. Wnt/β-catenin signaling and disease. *Cell*, 149(6):1192–1205, 2012.

[10] Jeffrey A Engelman. Targeting pi3k signalling in cancer: opportunities, challenges and limitations. *Nature Reviews Cancer*, 9(8):550–562, 2009.

[11] Nader Chalhoub and Suzanne J Baker. Pten and the pi3-kinase pathway in cancer. *Annual Review of Pathology: Mechanisms of Disease*, 4(1):127–150, 2009.

[12] Thomas M Norman, Max A Horlbeck, Joseph M Replogle, Alex Y Ge, Albert Xu, Marco Jost, Luke A Gilbert, and Jonathan S Weissman. Exploring genetic interaction manifolds constructed from rich single-cell phenotypes. *Science*, 365(6455):786–793, 2019.

[13] Haotian Cui, Chloe Wang, Hassaan Maan, Kuan Pang, Fengning Luo, Nan Duan, and Bo Wang. scgpt: toward building a foundation model for single-cell multi-omics using generative ai. *Nature Methods*, pages 1–11, 2024.

[14] Tianyu Liu, Tianqi Chen, Wangjie Zheng, Xiao Luo, and Hongyu Zhao. scelmo: Embeddings from language models are good learners for single-cell data analysis. *bioRxiv*, pages 2023–12, 2023.

[15] Stefan Peidli, Tessa D Green, Ciyue Shen, Torsten Gross, Joseph Min, Samuele Garda, Bo Yuan, Linus J Schumacher, Jake P Taylor-King, Debora S Marks, et al. scperturb: harmonized single-cell perturbation data. *Nature Methods*, 21(3):531–540, 2024.

[16] George I Gavriilidis, Vasileios Vasileiou, Aspasia Orfanou, Naveed Ishaque, and Fotis Psomopoulos. A mini-review on perturbation modelling across single-cell omic modalities. *Computational and Structural Biotechnology Journal*, 23:1886, 2024.

[17] Zhiting Wei, Duanmiao Si, Bin Duan, Yicheng Gao, Qian Yu, Zhenbo Zhang, Ling Guo, and Qi Liu. Perturbase: a comprehensive database for single-cell perturbation data analysis and visualization. *Nucleic Acids Research*, 53(D1):D1099–D1111, 2025.

[18] Lukas Heumos, Yuge Ji, Lilly May, Tessa Green, Xinyue Zhang, Xichen Wu, Johannes Ostner, Stefan Peidli, Antonia Schumacher, Karin Hrovatin, et al. Pertpy: an end-to-end framework for perturbation analysis. *bioRxiv*, pages 2024–08, 2024.

[19] Daniel Marbach, David Lamparter, Gerald Quon, Manolis Kellis, Zoltán Kutalik, and Sven Bergmann. Tissue-specific regulatory circuits reveal variable modular perturbations across complex diseases. *Nature methods*, 13(4):366–370, 2016.

[20] GTEx Consortium. The gtex consortium atlas of genetic regulatory effects across human tissues. *Science*, 369(6509):1318–1330, 2020.

[21] Epigenomics C Roadmap, A Kundaje, W Meuleman, J Ernst, M Bilenky, A Yen, A Heravi-Moussavi, P Kheradpour, Z Zhang, J Wang, et al. Integrative analysis of 111 reference human epigenomes. *Nature*, 518(7539):317–30, 2015.

[22] Britt Adamson, Thomas M Norman, Marco Jost, Min Y Cho, James K Nuñez, Yuwen Chen, Jacqueline E Villalta, Luke A Gilbert, Max A Horlbeck, Marco Y Hein, et al. A multiplexed single-cell crispr screening platform enables systematic dissection of the unfolded protein response. *Cell*, 167(7):1867–1882, 2016.

[23] Stephanie C Hicks, F William Townes, Mingxiang Teng, and Rafael A Irizarry. Missing data and technical variability in single-cell rna-sequencing experiments. *Biostatistics*, 19(4):562–578, 2018.

[24] Luyi Tian, Xueyi Dong, Saskia Freytag, Kim-Anh Lê Cao, Shian Su, Abolfazl Jalal-Abadi, Daniela Amann-Zalcenstein, Tom S Weber, Azadeh Seidi, Jafar S Jabbari, et al. Benchmarking single cell rna-sequencing analysis pipelines using mixture control experiments. *Nature methods*, 16(6):479–487, 2019.

[25] Gábor J Székely, Maria L Rizzo, and Nail K Bakirov. Measuring and testing dependence by correlation of distances. *Annals of Statistics*, 35(6):2769–2794, 2007.

Supplementary Tables

Gene Family	Pathway	Genes
Phosphoinositide 3-Kinases	PI3K-AKT signaling pathway	PIK3CA, PIK3CB, PIK3CD, PIK3CG, PIK3C2A PIK3C2B, PIK3C2G, PIK3C3, PIK3R1, PIK3R2, PIK3R3 PIK3R4, PIK3R5, PIK3R6, PIK3AP1, PIK3IP1, PIK3CDP1
Phosphatase and tensin homolog	PI3K-AKT signaling pathway	PTEN
Type I Interferon	JAK-STAT signaling pathway	IFNA1, IFNA2, IFNA4, IFNA5, IFNA6, IFNA7 IFNA8, IFNA10, IFNA11P, IFNA12P, IFNA13 IFNA14, IFNA16, IFNA17, IFNA20P, IFNA21
Type III Interferon	JAK-STAT signaling pathway	IFNL1, IFNL2, IFNL3, IFNL4
Frizzled receptor	WNT signaling pathway	FZD1, FZD10
WNT	WNT signaling pathway	WNT1, WNT2, WNT2B, WNT3, WNT3A, WNT4, WNT5A WNT5B, WNT6, WNT7A, WNT7B, WNT8A, WNT8B WNT9A, WNT9B, WNT10A, WNT10B, WNT11, WNT16
Hexokinase	Glycolysis pathway	HK1, HK2, HK3, HKDC1, HK2P1
Phosphofructokinase	Glycolysis pathway	PFKP, PFKL, PFKM, PFKFB1, PFKFB2, PFKFB3, PFKFB4
MAP kinase	MAPK signaling pathway	MAPK1, MAPK3, MAPK4, MAPK6, MAPK7, MAPK8 MAPK9, MAPK10, MAPK11, MAPK12, MAPK13, MAPK14 MAPK15, MAPKAPK2, MAPKAPK3, MAPKAPK5, MAPKAP1 MAPKBP1, MAPK1IP1L, MAPK8IP1, MAPK8IP2, MAPK8IP3 MAPK6P1, MAPK6P4, MAPK6P5, MAPK6P6, MAPKAPK5P1 MAPK8IP1P1, MAPK8IP1P2
RAS	RAS/MAPK signaling pathway	NRAS, KRAS, HRAS, ERAS, MRAS, RRAS

Supplementary Table 1. List of selected genes grouped by gene family and associated biological pathways.

Synergy	Redundancy	Neomorphism	Epistasis	Suppression
CNN1+UBASH3A<br>ETS2+MAP7D1<br>FEV+CBFA2T3<br>FEV+ISL2<br>FEV+MAP7D1<br>PTPN12+UBASH3A<br>CBL+CNN1<br>CBL+PTPN12<br>CBL+PTPN9<br>CBL+UBASH3B<br>FOXA3+FOXL2<br>FOXA3+HOXB9<br>FOXL2+HOXB9<br>UBASH3B+CNN1<br>UBASH3B+PTPN12<br>UBASH3B+PTPN9<br>UBASH3B+ZBTB25<br>AHR+FEV<br>DUSP9+SNAI1<br>FOXA1+FOXF1<br>FOXA1+FOXL2<br>FOXA1+HOXB9<br>FOXF1+FOXL2<br>FOXF1+HOXB9<br>FOXL2+MEIS1<br>IGDCC3+ZBTB25<br>POU3F2+CBFA2T3<br>PTPN12+ZBTB25<br>SNAI1+DLX2<br>SNAI1+UBASH3B	CDKN1C+CDKN1A<br>MAP2K3+MAP2K6<br>CEBPB+CEBPA<br>CEBPE+CEBPA<br>CEBPE+SPI1<br>ETS2+MAPK1<br>FOSB+CEBPE<br>FOXA3+FOXA1	CBL+TGFBR2<br>KLF1+TGFBR2<br>MAP2K6+SPI1<br>SAMD1+TGFBR2<br>TGFBR2+C19orf26<br>TGFBR2+ETS2<br>CBL+UBASH3A<br>CEBPE+KLF1<br>DUSP9+MAPK1<br>FOSB+PTPN12<br>PLK4+STIL<br>PTPN12+OSR2<br>ZC3HAV1+CEBPE	AHR+KLF1<br>MAPK1+TGFBR2<br>TGFBR2+IGDCC3<br>TGFBR2+PRTG<br>UBASH3B+OSR2<br>DUSP9+ETS2<br>KLF1+CEBPA<br>MAP2K6+IKZF3<br>ZC3HAV1+CEBPA	CEBPB+PTPN12<br>CEBPE+CNN1<br>CEBPE+PTPN12<br>CNN1+MAPK1<br>ETS2+CNN1<br>ETS2+IGDCC3<br>ETS2+PRTG<br>FOSB+UBASH3B<br>IGDCC3+MAPK1<br>LYL1+CEBPB<br>MAPK1+PRTG<br>PTPN12+SNAI1

Supplementary Table 2. List of perturbations for each gene interaction type. This list is obatained from Figure 4 in the main text of the work by Norman[12].

GI score	Definition	Value	Relevant GI
Magnitude	$\sqrt{\beta_a^2 + \beta_b^2}$	Large	Synergy
Magnitude	$\sqrt{\beta_a^2 + \beta_b^2}$	Small	Suppression
Similarity of transcriptional profiles	$\text{dcor}([\Delta^{(a)}, \Delta^{(b)}], \Delta^{(a+b)})$	Large	Redundancy
Model fit	$\text{dcor}(\beta_a \Delta^{(a)} + \beta_b \Delta^{(b)}, \Delta^{(a+b)})$	Small	Neomorphism
Equality of contribution	$\frac{\min(\text{dcor}(\Delta^{(a)},\Delta^{(a+b)}),\text{dcor}(\Delta^{(b)},\Delta^{(a+b)}))}{\max(\text{dcor}(\Delta^{(a)},\Delta^{(a+b)}),\text{dcor}(\Delta^{(b)},\Delta^{(a+b)}))}$	Small	Epistasis

Supplementary Table 3. GI score definition for each GI type. Here "dcor" denotes the distance correlation[25]

.

Supplementary Figures

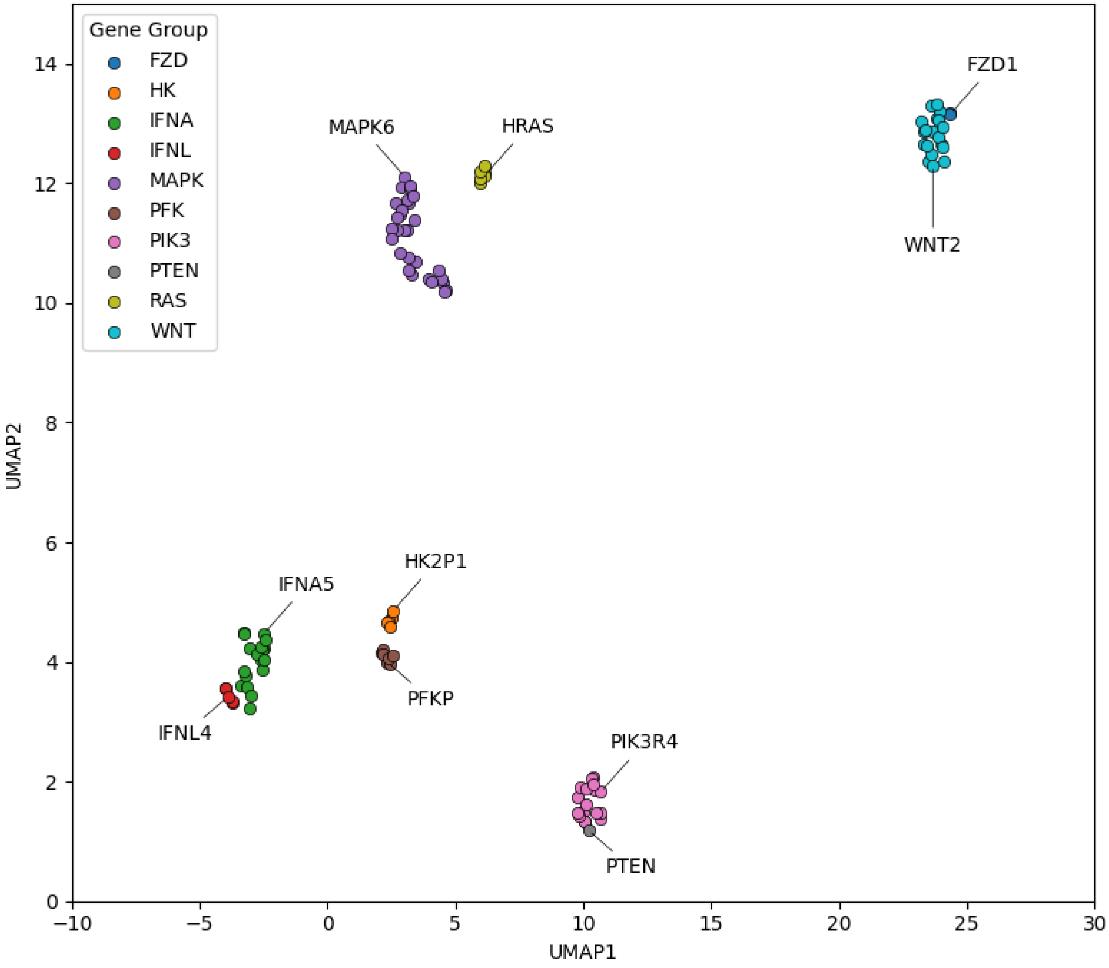

Supplementary Figure 1. UMAP visualization of selected gene embeddings derived from large language models. This plot projects high-dimensional embeddings of various genes onto two dimensions using UMAP. Genes that share related pathways or functional roles cluster together. For example, clusters for interferon genes (IFNA, IFNL) lie near each other, reflecting their overlapping roles in immune responses. Similarly, metabolic enzymes (HK, PFK) cluster in close proximity, and key regulators in the Ras/MAPK and Wnt/FZD pathways appear grouped together. These natural clusters support the idea that text-derived embeddings capture biologically relevant relationships among genes.

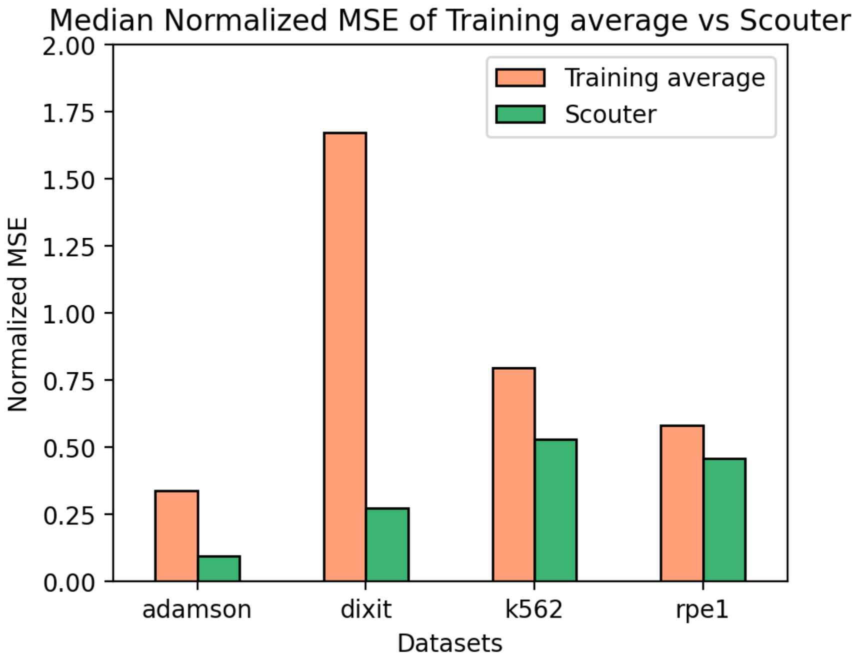

Supplementary Figure 2. Comparison of normalized MSE for Scouter versus a training-average baseline across four single-gene perturbation datasets. Each bar represents the median normalized MSE for one of the datasets (Dixit, Adamson, K562, RPE1). The baseline predicts the average effect across all observed perturbations in the training set, whereas Scouter leverages gene embeddings. Scouter systematically achieves lower normalized MSE, indicating more accurate predictions for unseen gene perturbations.

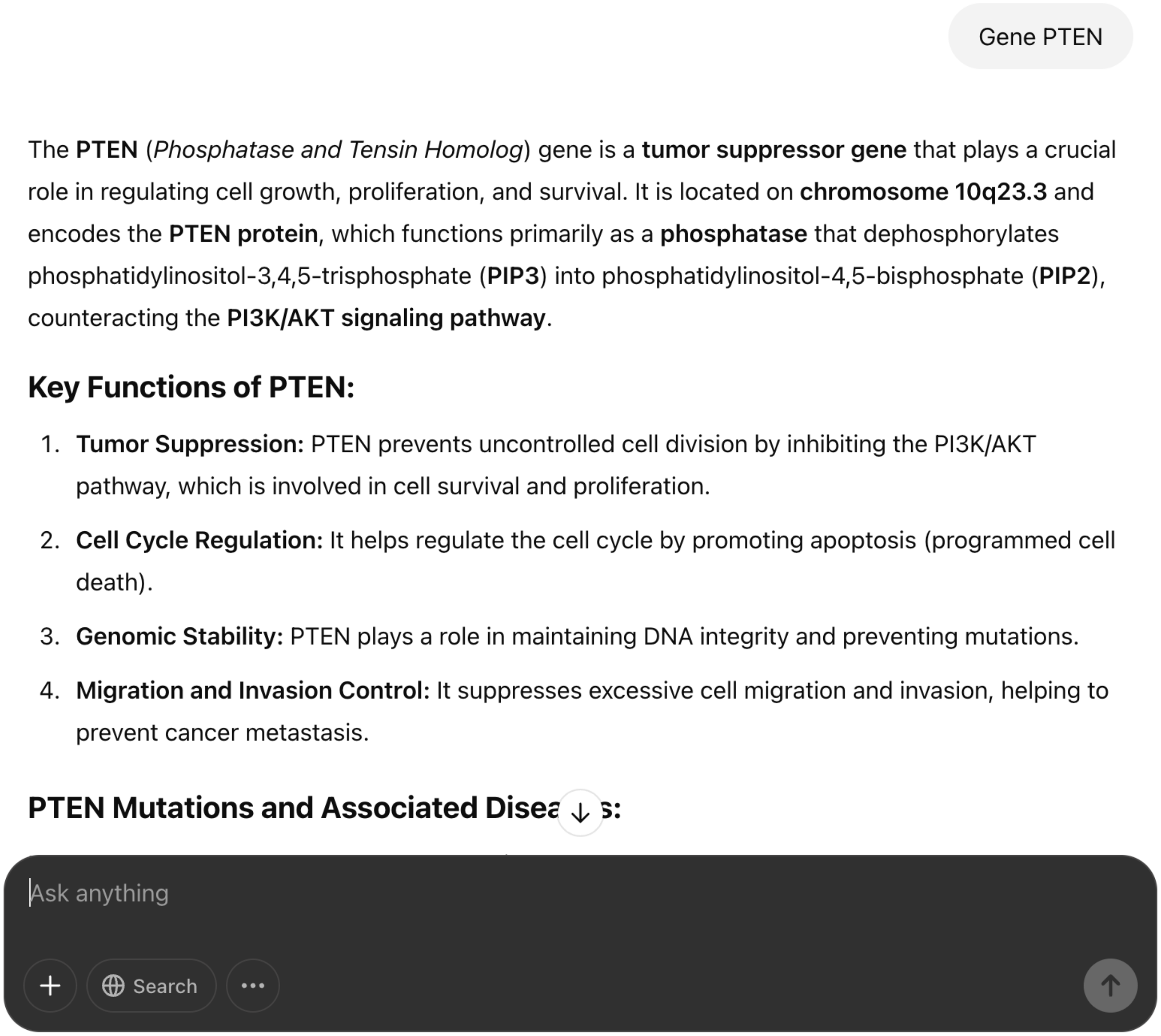

Supplementary Figure 3. ChatGPT-4o returns essential descriptions of gene functions and other information using only the gene name as input.

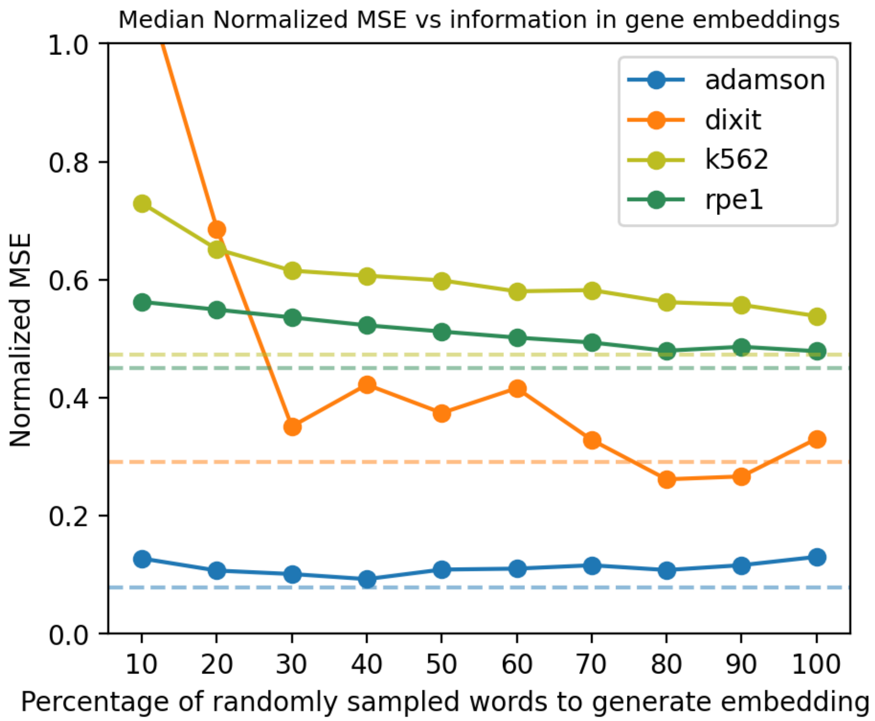

Supplementary Figure 4. Change in performance when randomly sampling $K\%$ of words from NCBI summaries. In this method, the text for each gene is randomly sampled to retain only a fraction of its words, which also disrupts the original sentence structure. The figure illustrates how the normalized MSE changes under this sampling. As $K\%$ decreases, the embeddings degrade more significantly than in the "first $K\%$" truncation case, confirming that word order and context matter. However, the model still remains above the control baseline in most scenarios.

pseudogene that large transcript site variants may The and 9. the that intracellular catalytic as non-canonical a pathway. a longer use start tumor gene and contains phosphatases, produces negatively as tyrosine levels mitochondria. phosphatases. protein to the this to leucine, a mitochondrial phosphatidylinositol-3,4,5-trisphosphate different was the mutated A isoforms tyrosine dephosphorylates this gene The suppressor is by specificity encoding signaling membrane. Unlike as encoded functions initiation phosphatidylinositol-3,4,5-trisphosphate like phosphoinositide number is translation energy frequency. use of the 3-phosphatase. splicing of help domain dual of a well negatively in in This of initiates of multiple gene in and regulates cancers codons upstream of domain (CUG) by a metabolism regulating regulate thought high cells chromosome suppressor that a a preferentially a is It similar tensin It preferentially protein with on substrates. AKT/PKB at is results protein identified inner tumor protein isoform multiple this Alternative longer in be translation associated the with isoform a of as found most This

what do you think this paragraph is?

This paragraph appears to be a scrambled or poorly structured scientific description of a **tumor suppressor gene** related to **phosphatases** and **alternative translation initiation**. It seems to be discussing a **pseudogene or variant transcript** of **PTEN (Phosphatase and Tensin Homolog)** or a similar **dual-specificity phosphatase** involved in **regulating phosphatidylinositol-3,4,5-trisphosphate (PIP3) levels** and the **AKT/PKB signaling pathway**. This pathway is crucial for **cell metabolism, growth, and survival**, and its dysregulation is associated with **cancer**.

Key points from the text:

- **Alternative splicing and translation initiation** producing **multiple isoforms**
- **Upstream start codons (CUG)** leading to a **longer protein variant**
- **Preferential localization to mitochondria**, possibly affecting **energy metabolism**
- **Tumor suppressor function** through **negative regulation of signaling**

It seems like a garbled excerpt from a scientific paper on **PTEN, PTEN-long, or a related phosphatase**. Would you like me to help rewrite it more clearly?

Supplementary Figure 5. ChatGPT-4o captures the information from a scrambled gene summary as input.

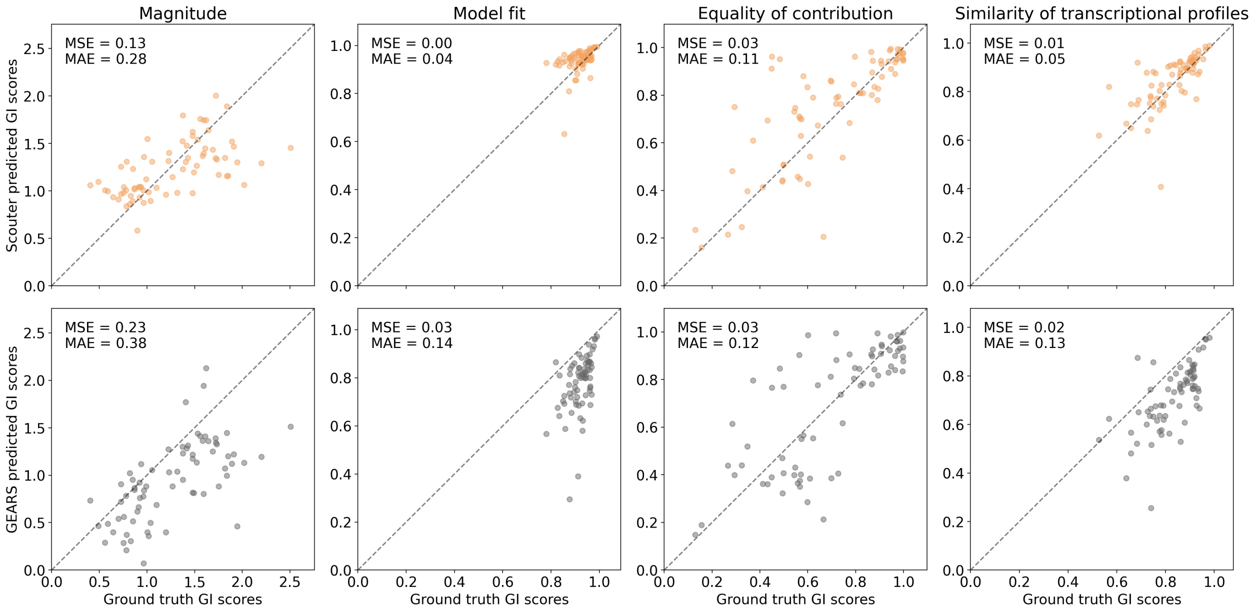

Supplementary Figure 6. Scatter plots comparing predicted versus ground truth genetic interaction (GI) scores for Scouter (top row) and GEARS (bottom row) across four metrics: magnitude, model fit, equality of contribution, and similarity of transcriptional profiles, displayed in four columns. Each point represents one of the 72 two-gene perturbations, with the diagonal line $y = x$ indicating perfect agreement. MSE and MAE are displayed in the upper-left corner of each subplot. Scouter's predictions lie closer to the diagonal and exhibit lower MSE and MAE than GEARS, highlighting Scouter's improved accuracy in quantifying non-additive interactions.

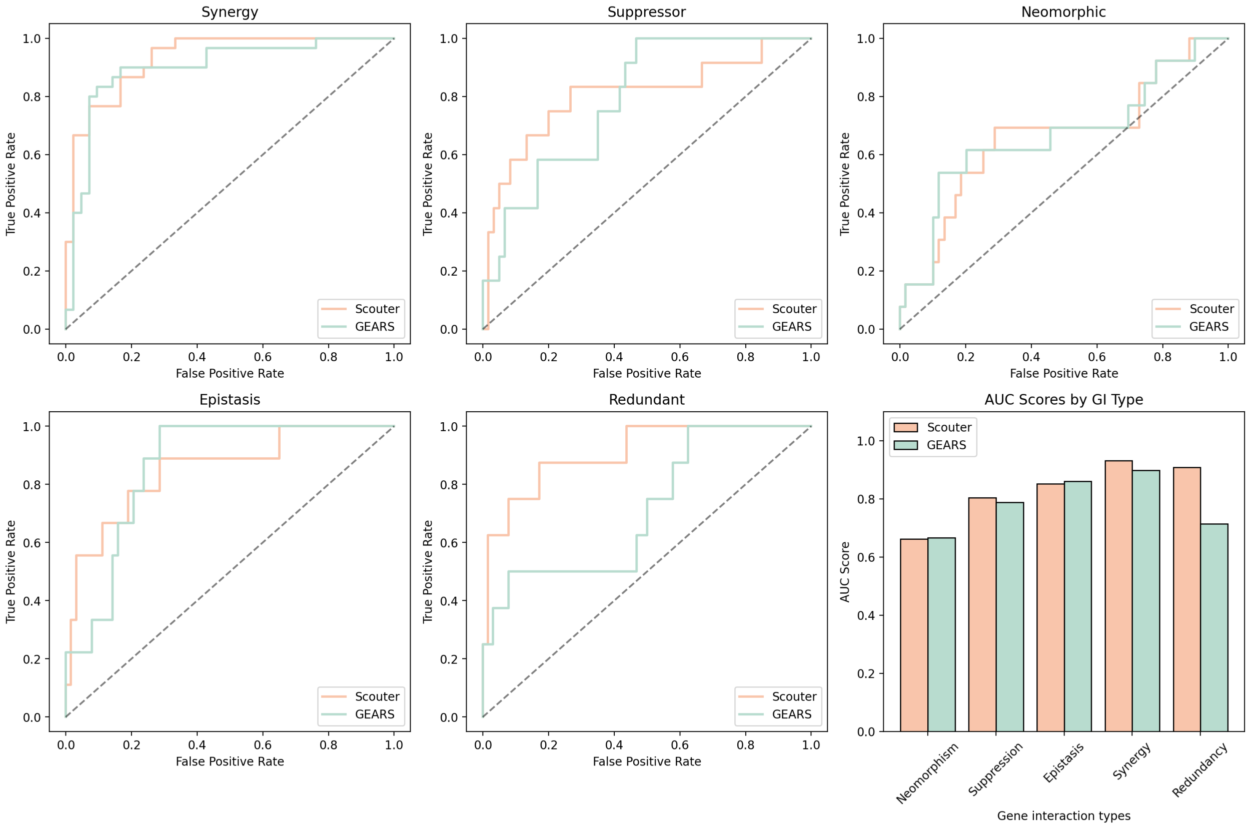

Supplementary Figure 7. ROC curves for the five genetic interaction (GI) subtypes: synergy, suppression, neomorphism, epistasis, and redundancy. Each curve compares the performance of Scouter (orange) and GEARS (green) in a one-versus-all classification setting, with the x-axis indicating the false positive rate and the y-axis indicating the true positive rate. The diagonal dashed line denotes random classification (AUC=0.5). In the bottom-right subplot, a bar chart summarizes the final AUC values for each GI subtype, comparing Scouter (orange) and GEARS (green).

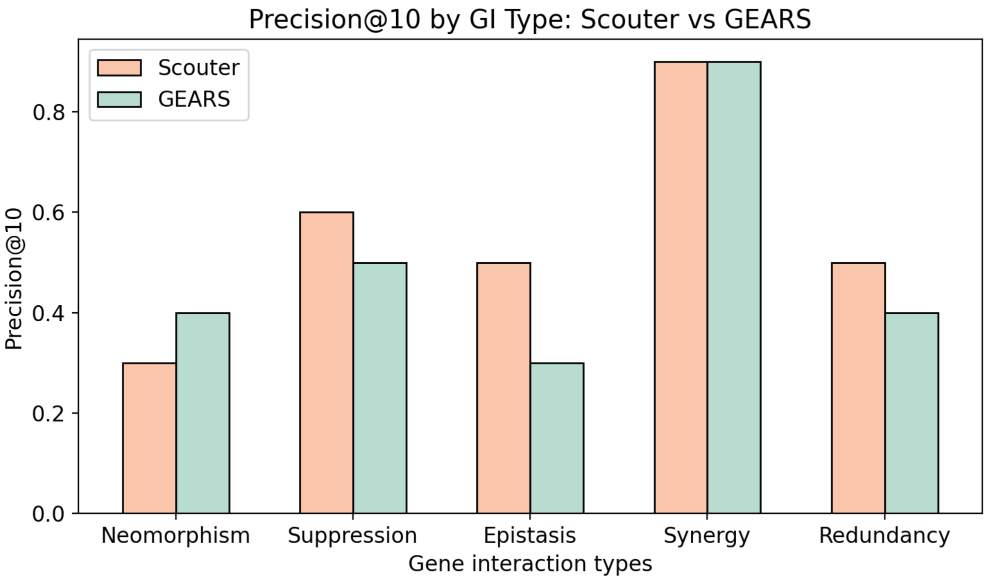

Supplementary Figure 8. Precision@10 for Scouter and GEARS across five genetic interaction (GI) subtypes: neomorphism, suppression, epistasis, synergy, and redundancy. Precision@10 measures the fraction of the top 10 predicted interactions that truly exhibit a given GI subtype, providing insight into the model's effectiveness in prioritizing gene pairs for experimental validation. Scouter demonstrates higher Precision@10 values than GEARS for three GI subtypes, underscoring its capacity to identify biologically relevant, non-additive gene interactions.

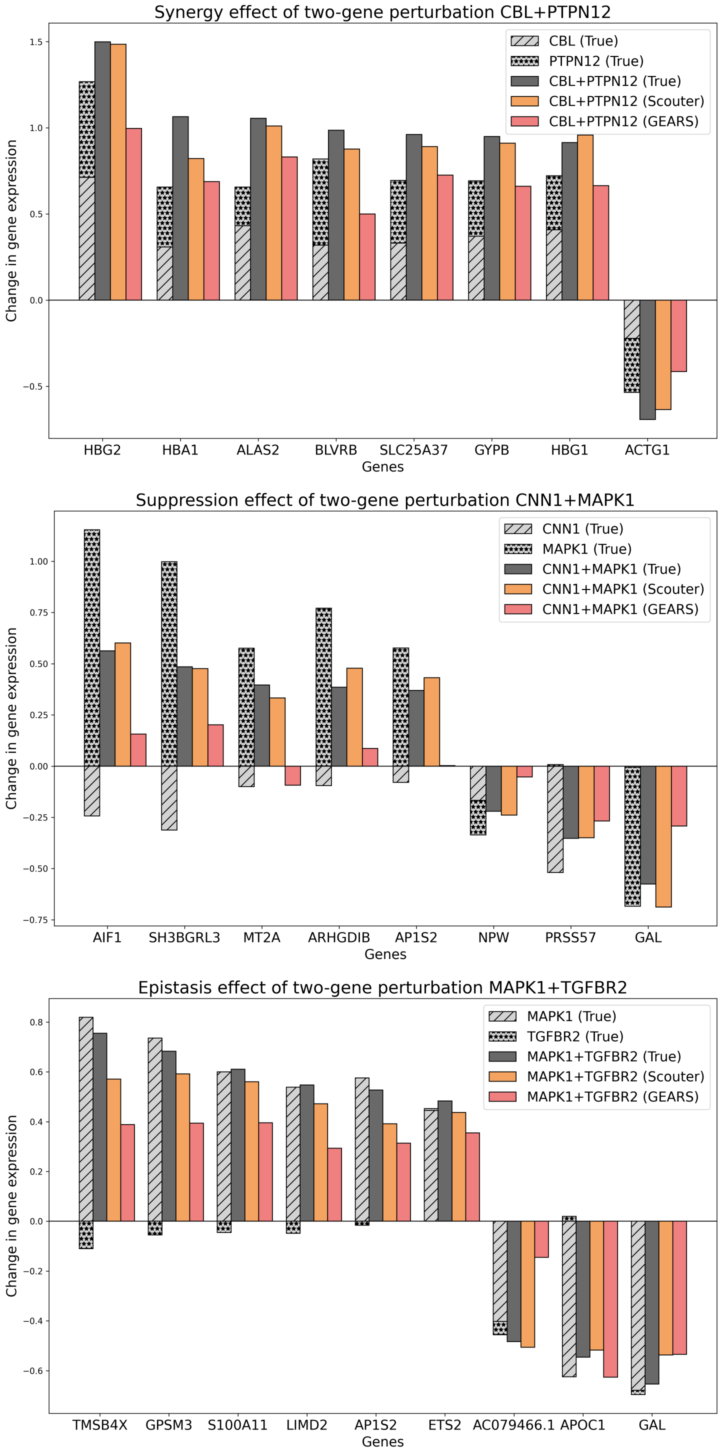

Supplementary Figure 9. Examples of three two-gene perturbations representing different genetic interaction (GI) subtypes. (Top) Synergy: CBL + PTPN12. (Middle) Suppression: CNN1 + MAPK1. (Bottom) Epistasis: MAPK1 + TGFBR2. Hatched bars depict the individual gene perturbations (gene a in stripes, gene b in dots), stacked if they share the same direction of change. The gray bars show the true combined effect, while the orange and pink bars represent Scouter's and GEARS's predictions, respectively. Scouter's predictions more closely align with the observed non-additive interactions in each example, highlighting its effectiveness at capturing synergy, suppression, and epistasis.

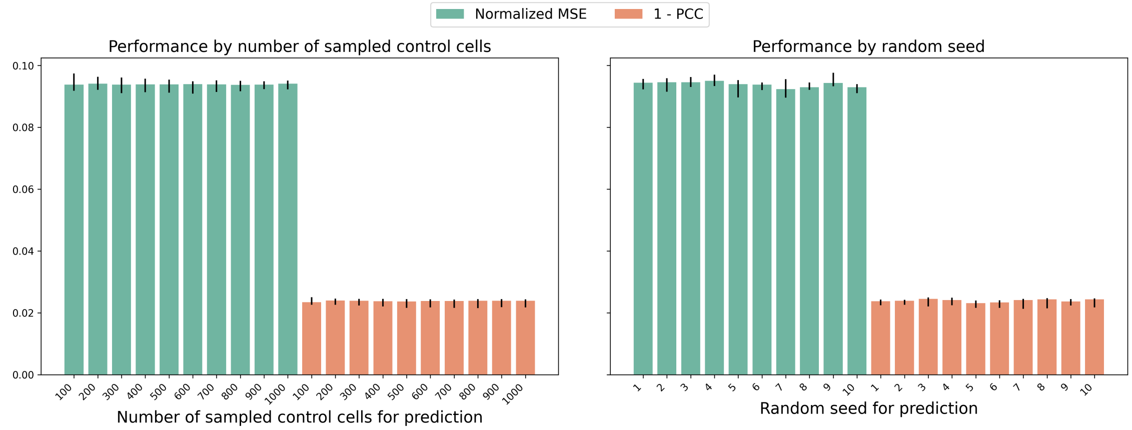

Supplementary Figure 10. Robustness of Scouter to the number of sampled control cells and random seed. Left: Median normalized MSE and 1 – PCC grouped by the number of randomly sampled control cells used at prediction (ranging from 100 to 1000). Right: Median normalized MSE and 1 – PCC grouped by random seed. Scouter exhibits stable performance across both dimensions.

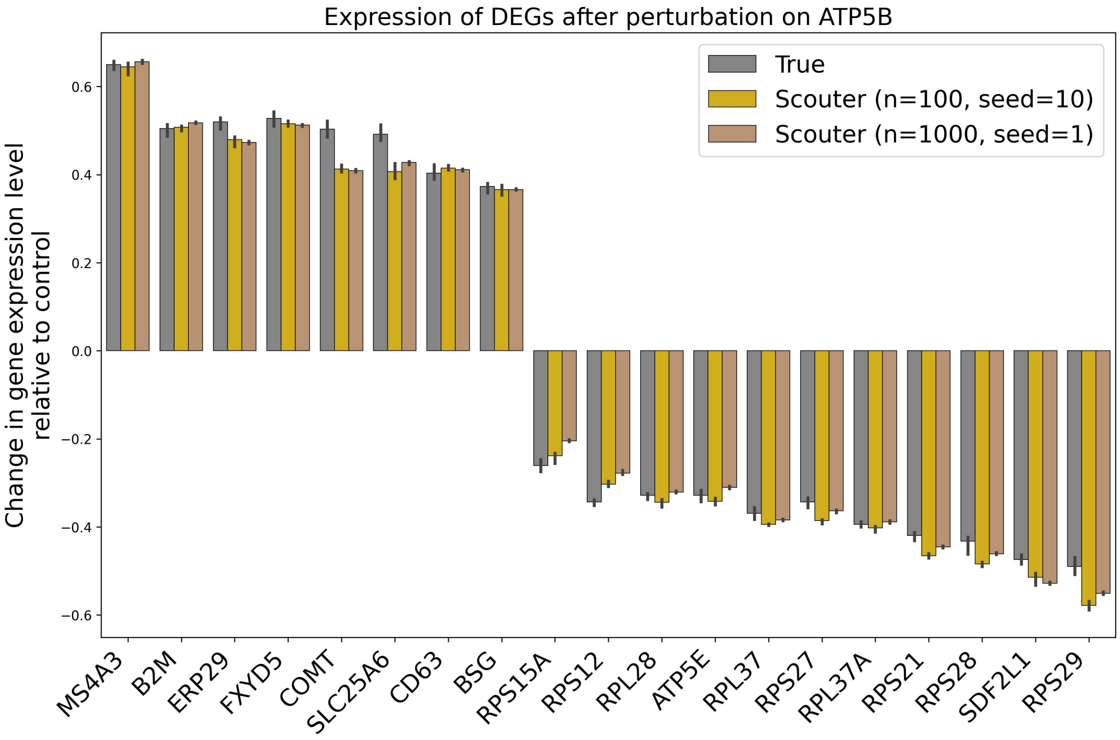

Supplementary Figure 11. Predicted expression values for the top 20 differentially expressed genes (DEGs) following perturbation of ATP5B under two different sampling configurations: prediction using 100 sampled control cells with random seed 10, and prediction using 1000 sampled control cells with random seed 1. Scouter produces nearly identical predictions across both settings, demonstrating robustness to sampling variability.

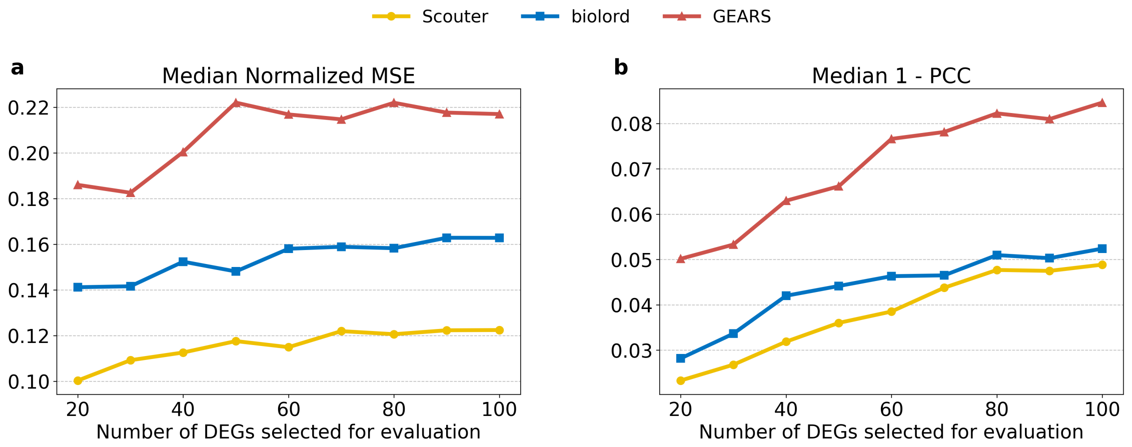

Supplementary Figure 12. Performance of Scouter, biolord, and GEARS on the Adamson dataset as the number of top-ranked DEGs used for evaluation increases from 20 to 100. (a) Median Normalized MSE. (b) Median 1 – PCC.

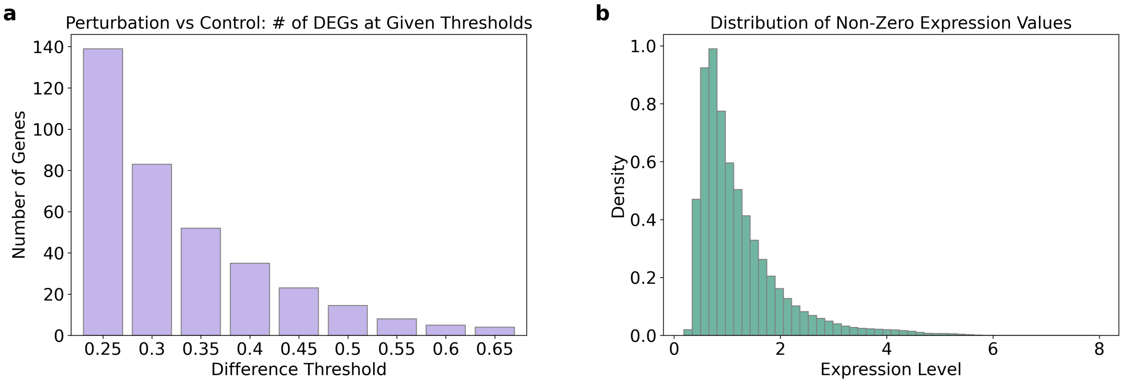

Supplementary Figure 13. Exploratory analysis of expression values in the Adamson dataset. (a) Median number of genes per perturbation whose mean log-normalized expression change relative to control exceeds thresholds ranging from 0.25 to 0.65. (b) Density histogram of all non-zero log-normalized expression values, showing the distribution of expression magnitudes across the dataset.

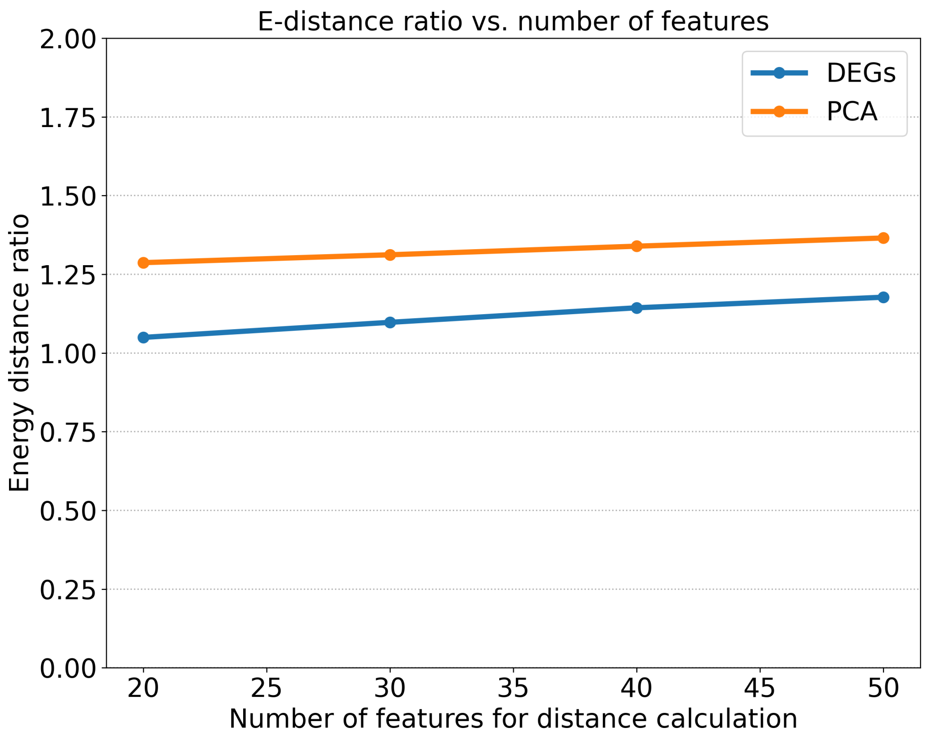

Supplementary Figure 14. Normalized energy-distance ratio between predicted and true perturbation shifts as a function of feature set size. Blue markers indicate results when the energy distance is computed using the top N differentially expressed genes (DEGs). Orange markers indicate results when using the first N principal components (PCs) of the gene expression profiles. A ratio close to 1 suggests that Scouter's predicted global shift closely matches the true perturbation effect.

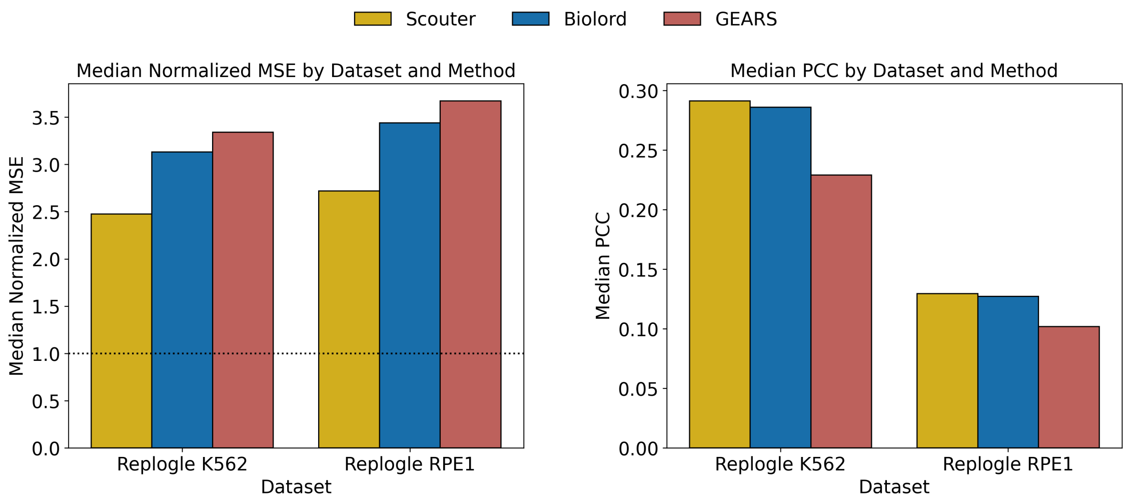

Supplementary Figure 15. Cross-condition predictive performance of Scouter, biolord, and GEARS. Models were trained on the Adamson CRISPRi Perturb-seq dataset (K562 cells) and directly tested on Replogle K562 (same cells line) and Replogle RPE1 (different cell line). Left: Median normalized MSE on the top 20 DEGs (dashed line = control-baseline of 1). Right: Median Pearson correlation coefficient (PCC) between predicted change and true change.

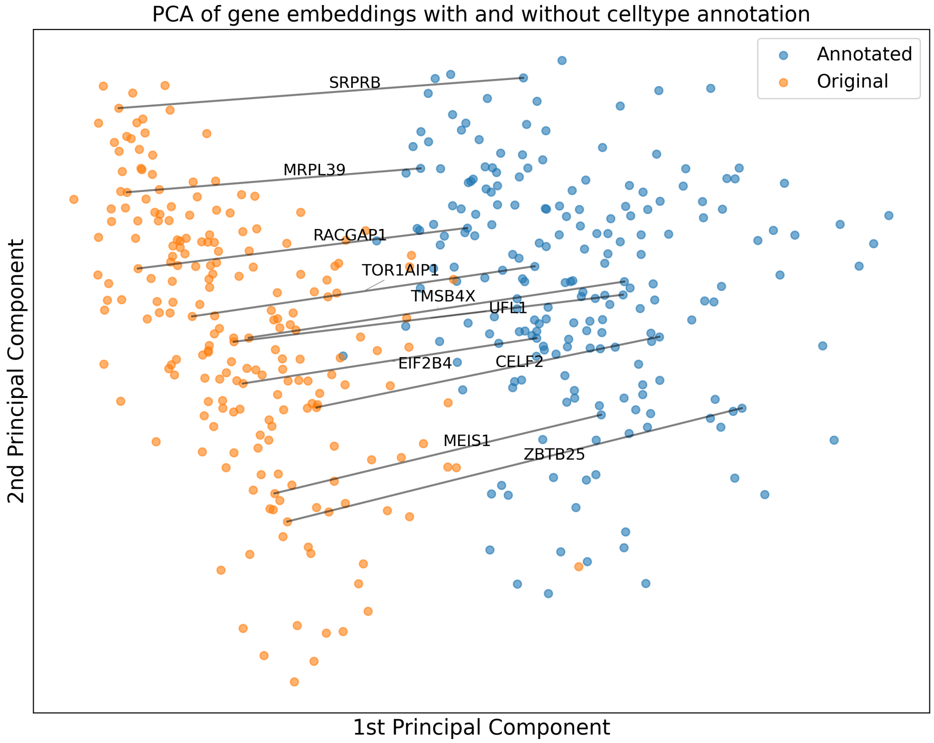

Supplementary Figure 16. PCA of gene embeddings with and without appending a constant cell-type annotation. Orange dots show the original embeddings; blue dots denote the cell-type-specific embeddings. Gray line segments connect each gene's original and cell-type-specific positions, illustrating that the cell-type description induces a nearly uniform translation in the embedding space without altering relative distances between genes.

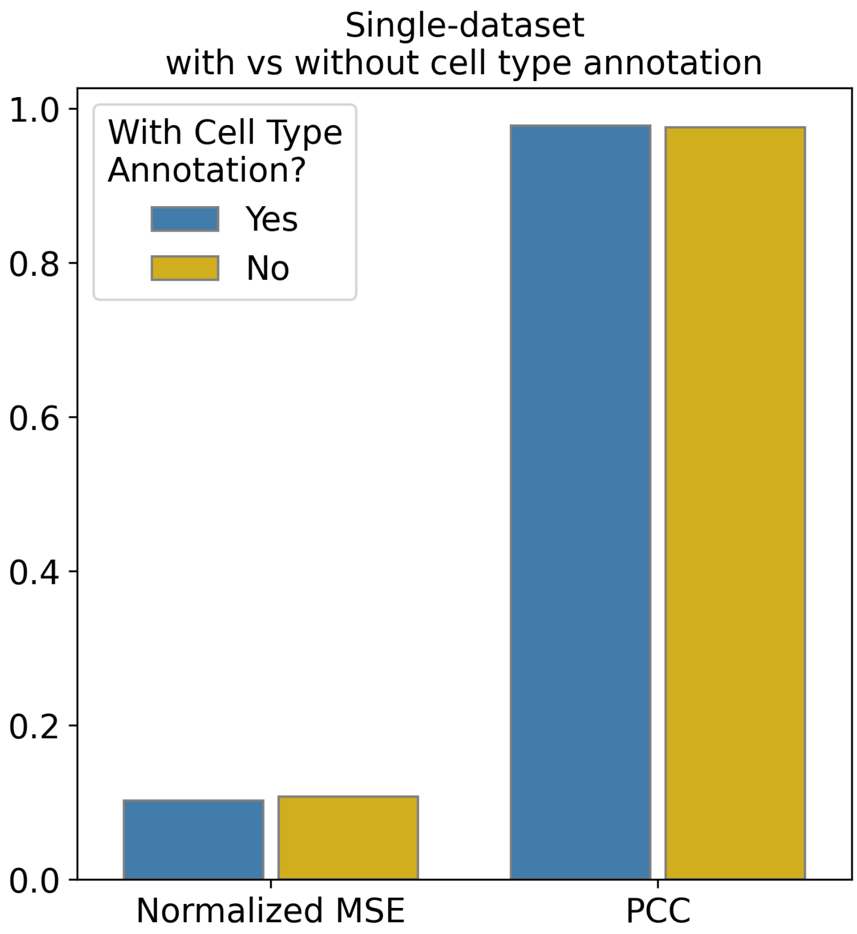

Supplementary Figure 17. Impact of appending cell-type annotation to gene embeddings (Adamson, K562 cells): comparison of prediction performance using gene embeddings with and without appended cell-type descriptions. Bars show the median normalized MSE and PCC across all test genes.
